# Trade-offs for climate-resilient pastoral livelihoods in wildlife conservancies in the Mara ecosystem, Kenya

**DOI:** 10.1186/s13570-017-0085-1

**Published:** 2017-05-23

**Authors:** Claire Bedelian, Joseph O. Ogutu

**Affiliations:** 1 International Livestock Research Institute, P.O. Box 30709-00100, Nairobi, Kenya; 2 Overseas Development Institute, 203 Blackfriars Road, London, SE1 8NJ UK; 3 Anthropology Department, University College London, 14 Taviton Street, London, WC1H 0BW UK; 4 Institute of Crop Science-340, University of Hohenheim, 70599 Stuttgart, Germany

**Keywords:** Conservancies, Kenya, Pastoral livelihoods, Livestock grazing, Livestock trends, Maasai Mara

## Abstract

Pastoralists in the wildlife-rich East African rangelands use diversification into conservation and tourism as a strategy to supplement livestock-based livelihoods and to spread risk. Tourism incomes are an important alternative source during drought, when livestock incomes decline. However, tourism may also reduce access to rangeland resources, and an abundant wildlife may destroy crops and injure, kill or transmit disease to livestock or people. This paper investigates the ability of wildlife conservancies in the Mara, Kenya, to act as an alternative for pastoralists that mitigates risks and maintains resilience in a changing climate. It analyses data to examine how conservancies contribute to and integrate with pastoral livelihoods, and to understand how pastoralists are managing their livestock herds in response to conservancies. It finds conservancy payments can provide an important, reliable, all-year-round source of income and prevent households from selling their animals during stress and for cash needs. Conservancies also retain grass banks during the dry season and provide opportunities for pastoralists to access good-quality forage. However, they reduce access to large areas of former grazing land and impose restrictions on livestock mobility. This affects the ability of pastoralists to remain flexible and able to access seasonally variable resources. Conflicts between grazing and conservancies may also heighten during drought times. Furthermore, income from land leases is not more than the contribution of livestock, meaning conservancy land leases create trade-offs for livestock-based livelihoods. Also, income is based on land ownership, which has inequity implications: women and other marginalised groups are left out.

**Electronic supplementary material:**

The online version of this article (doi:10.1186/s13570-017-0085-1) contains supplementary material, which is available to authorized users.

## Introduction

Pastoralism is a production system adapted to arid and semi-arid lands. Pastoralists have long-standing traditions and strategies of using resources, characterised by mobility, flexibility, adaptability and reciprocity (Fernandez-Gimenez and Le Febre 2006). These strategies allow pastoralists to adapt to the variability in rangeland resources and climate that is inherent in these systems (Scoones 1994). Although pastoralism has evolved to manage climate variability through mobility and risk-spreading strategies, the climate in East Africa is becoming increasingly variable and unpredictable. Climate change is expected to increase the frequency and severity of extreme events and shocks, such as droughts and floods (Field et al. 2014). Predictions for East Africa show a likely warming by 2 °C and increases in annual precipitation and extreme rainfall events (IPCC 2014). It is expected that climate change will modify the way people cope with variability in their resources. These climatic changes are likely to require pastoralists to develop new adaptation, risk management and coping strategies.

Furthermore, the effects of climate change are likely to compound those of other transformations going on in pastoral systems, including poverty, human and livestock population growth, conflict, competition for land and rangeland fragmentation (Behnke 2008; Galvin 2009; Reid et al. 2014). Economic, policy and institutional drivers cause fragmentation, resulting in land-use change, habitat modification and land subdivision (Galvin et al. 2008; Hobbs et al. 2008). Fragmentation restricts access to resources and increases vulnerability to stresses and shocks. Climate change and fragmentation will interact with one another, such that increasing fragmentation will restrict the flexibility and mobility of pastoralists in the wake of more frequent floods and droughts on the rangelands (Galvin 2009).

It is argued, however, that, despite the challenges climate change pose to pastoralists, pastoralism is a land use already adapted to variability in rainfall and thus offers better adaptation potential than other competing land uses (Nori and Davies 2007). Pastoralists have long-standing traditional strategies and strong social institutions for using resources and responding to climate variability. They use mobility to track variable and unpredictable resources and are thus better able to respond to and cope with drought. Mobile pastoralists do better than sedentary ones during drought and are less likely to lose stock (Little et al. 2008). Facilitating mobility will thus help ensure continued resilience of pastoral livelihoods in a future changed climate. Resilient livelihoods will be those best able to cope with the increased climatic shocks in these systems.

Diversification into viable alternative livelihoods is another way to spread risk and is likely to become increasingly important as climate changes. As fragmentation constrains mobility and access to key resources, pastoralists have to increasingly rely on non-livestock sources of income for their livelihoods (Homewood et al. 2009). Diversification of pastoral livelihoods is widely observed across pastoralists in East Africa (Homewood et al. 2009; Kristjanson et al. 2002; Little et al. 2001). Diversification into tourism is a potential option in areas with high wildlife abundance and a high tourism volume. The majority of Kenya’s wildlife is found within its arid and semi-arid lands that cover 88% of Kenya’s land surface, so pastoral lands are vital habitats for wildlife and tourism. In the pastoral savannas of East Africa, wildlife and livestock have lived side by side for millennia, and traditional pastoralism has been considered to be compatible with wildlife conservation (Western 1982; Homewood and Rodgers 1991; McCabe and Perkin 1992). Pastoralism keeps rangelands open and, when practised on common land, slows down the fragmentation of land and creates synergies for wildlife. Pastoralists thus play an important role in maintaining these landscapes, wildlife populations and hotspots of biodiversity (Homewood 2008; Nelson 2012; Reid 2012). Synergies between pastoralism and wildlife also highlight their shared potential as adaptive land uses in response to climate change in the erratic and variable East African savannas.

There are thus synergies, and a level of compatibility, in combining pastoralism and tourism. However, there can also be trade-offs: tourism interventions may alter how pastoralists are able to access rangeland resources; wildlife may eat and destroy crops and injure, kill or transmit diseases to livestock or people. Benefits from tourism may thus be unable to compensate for the opportunity costs of living with wildlife and the loss of established livelihood activities (Ferraro 2002; Norton-Griffiths and Southey 1995). Furthermore, the negatives may become more marked in the context of climate change if resilient and flexible livelihoods become more important.

There is also little evidence that tourism has benefited pastoralists: tourism incomes accruing to pastoralists have typically been small and few pastoralists derive their main income from tourism (DeLuca 2004; Homewood et al. 2009; Sachedina 2008). Moreover, tourism revenues tend to be inequitably distributed, with the wealthier and better placed individuals capturing most revenues (Homewood et al. 2009; Thompson and Homewood 2002). Benefits also tend to be spread unevenly along age, gender, educational, race and ethnic lines (DeLuca 2004). Furthermore, tourism can be a risky livelihood alternative to pastoralism, because it is sensitive to political instability, economic downturns, insecurity and epidemics. For these reasons, pastoralists rarely view tourism as a substitute for their livestock-based activities, but rather as a complement to them (DeLuca 2004; Homewood et al. 2009). As a supplementary activity, tourism can be a less risky addition.

In Kenya, tourism is one of the top earners of foreign exchange, generating almost $1 billion in income in 2015 (exchange rate US $1 = 90 KES in 2015) (KNBS 2015). Tourism is also an important contributor to Kenya’s national gross domestic product (GDP) (World Bank 2011). Sixty-five percent of Kenya’s large mammal wildlife lives outside of formally protected areas and within community and privately owned pastoral rangelands (Western et al. 2009). It is these areas that are becoming the increasing focus of many new conservation and tourism development initiatives.

A growing number of wildlife conservancies are being set up on community and private pastoral lands with a long history of mixed livestock and wildlife use (Kaelo 2013). In Kenya’s Wildlife Conservation and Management Act of 2013, a conservancy is defined as ‘land set aside by an individual landowner, body corporate, group of owners or a community for purposes of wildlife conservation’. By the end of 2015, there were 178 conservancies in Kenya: 120 that were established and 58 that were emerging (Reid, RS, D Kaelo, KA Galvin, and R Harmon: Pastoral wildlife conservancies in Kenya: A bottom-up revolution in conservation, balancing livelihoods and conservation?, unpublished). The Kenya Wildlife Conservancies Association distinguishes three types of conservancies: private conservancies set up by a private individual or corporate on private land, community conservancies set up by a community on community land and group conservancies created by pooling land by contiguous private land owners (King et al. 2015).

A survey of 57 conservancy managers from conservancies across Kenya found the main aims in setting up conservancies to be improved incomes for local people and conservation of habitat for wildlife and tourism (Reid, RS, D Kaelo, KA Galvin, and R Harmon: Pastoral wildlife conservancies in Kenya: A bottom-up revolution in conservation, balancing livelihoods and conservation?, unpublished). Incomes from tourism are especially important during drought times, and the potential of conservancies as a drought-coping and risk mitigation strategy has been argued (Osano et al. 2013). However, although the extra source of revenue represents an important supplementary income, restrictions on land use that new tourism uses apply can create trade-offs as traditional livelihood activities are curtailed. This can affect the ability of pastoralists to access sufficient resources and maintain resilient livelihoods.

Using as a case study the Mara in Kenya, where a number of wildlife conservancies now exist, this paper seeks to explore these trade-offs to understand how conservation and tourism may be enhancing or restricting climate-resilient pastoral livelihoods. It looks at the ability of conservancies to serve as an alternative livelihood opportunity for pastoralists that mitigates risk and maintains resilience in a changing climate. Specifically, the paper asks:

1. What is the contribution of conservancies to pastoral livelihoods, relative to other livelihood activities?
2. How have household livestock-grazing strategies been altered as a result of restrictions on livestock grazing within conservancies?
3. What is the impact of conservancies on livestock density, distribution and composition?

The research helps elucidate the mechanisms through which pastoralists are managing their livestock herds to cope with shrinking pastoral ranges and reduce their vulnerability to drought and climate change risks. Results can inform better future conservation and tourism investments aimed at maintaining and enhancing pastoral resilience, as well as promoting wildlife conservation.

## Study area

**Figure 1:**
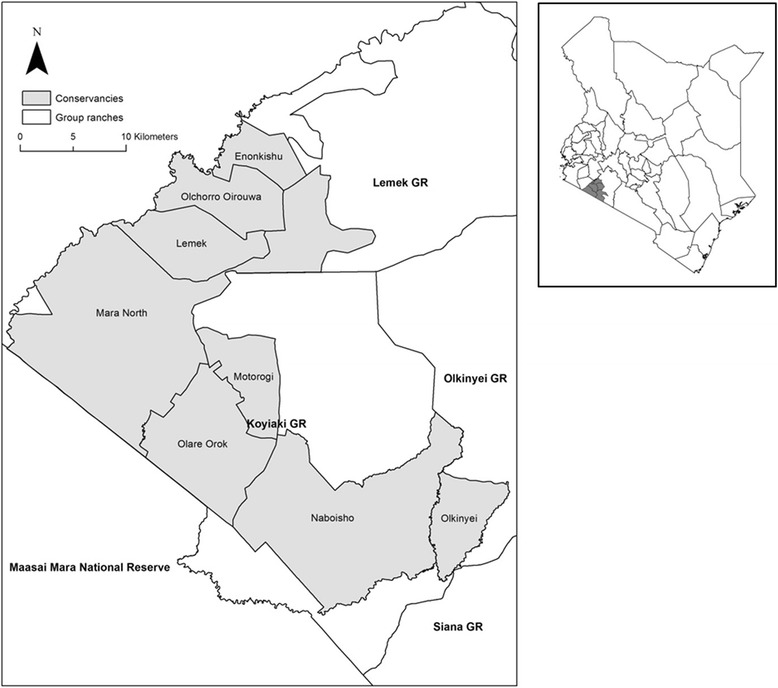
The Mara area, showing the MMNR, the surrounding group ranches and conservancies. Our study area focused on Koyiaki Group Ranch which is made up of the Mara North, Olare Motorogi and Naboisho Conservancies, and surrounding community areas shown in *white*

The Mara comprises the Maasai Mara National Reserve (MMNR) and surrounding conservancies and group ranches (Figure 1). The MMNR (1,530 km^2^) is a nationally protected area situated on the international border between Kenya and Tanzania’s Serengeti National Park. The MMNR (latitudes 1° 00′ to 2° 00′ S and longitudes 34° 45′ to 36° 00′ E) has the highest density of wildlife in Kenya, many of which spill out into and graze in neighbouring conservancies and group ranch lands during the wet season (Ogutu et al. 2011). As well as supporting a number of resident wildlife species, the Mara area provides dry season grazing and permanent water for the migratory wildebeest (*Connochaetes taurinus*), zebra (*Equus quagga*) and Thomson’s gazelle (*Gazella thomsoni*) as they move north from the Serengeti (Stelfox et al. 1986).

The Mara has two rainy seasons, with the short rains occurring during November to December and the long rains from March to June. Often, the short and long rains merge into one season, or the short rains may fail completely. Mean annual rainfall increases from the drier south-east (877 mm/year) to the wetter north-west (1,341 mm/year) (Ogutu et al. 2011). In recent decades, the Mara has experienced recurrent droughts, with particularly severe rainfall deficits occurring in 1984, 1993, 1999 to 2000, 2005 to 2006 and 2008 to 2009 (Ogutu et al. 2008a; 2011).

As in other rangeland areas in Kenya, the Mara is undergoing a transformation in land ownership from communal to individual landholdings. Government policies encouraged the privatisation and commercialisation of pastoral lands, driven by economic incentives to intensify livestock production. Land was demarcated in group ranches, which were owned and held under private title by a group of registered members, and managed by an elected committee (Galaty 1992). The expectation was that these would provide tenure security and thus create incentives for the Maasai to invest in range and breed improvement and reduce the tendency to accumulate large numbers of perceived low-quality indigenous breed livestock (Kimani and Pickard 1998).

Dissatisfaction with corrupt group ranch committees, dilution of individual shareholding as population size increased and a desire for security of tenure led group ranch members to push for the subdivision of group ranches (Galaty 1999; Homewood et al. 2004; Mwangi 2007a; Seno and Shaw 2002; Thompson and Homewood 2002). In the Mara, many group ranches are now subdivided, with individual land parcels allocated to male group ranch members. In many areas, this has been a long and contentious process, fraught with conflict, inequality and land-grabbing (Homewood et al. 2004; Mwangi 2007b; Thompson and Homewood 2002; Thompson et al. 2009).

The Mara has long been a leader in Kenya’s tourism industry, and the MMNR is one of Kenya’s top most visited protected areas. In 2011, the MMNR management plan estimated that predicted revenues accruing from the MMNR to the then two county councils responsible for its administration totalled more than $41 million annually (NCC and TMCC 2011). However, despite this tourism potential, local communities have not always gained fair or substantial benefit (Norton-Griffiths et al. 2008; Thompson et al. 2009). Various attempts to distribute tourism revenues to local communities have been beset by problems of mismanagement, unaccountability and inequality (Thompson and Homewood 2002; Thompson et al. 2009). A multi-site study in Maasailand (Homewood et al. 2009) shows that, although households in the Mara do receive the most from wildlife (approximately 20% of total annual household income) compared with similar sites in Kenya and Tanzania, even here, it is the wealthiest households that capture the greatest portion of revenues (Homewood et al. 2012; Thompson et al. 2009).

Since 2005, a number of conservancies have been set up on the communal and privatised group ranches adjacent to the MMNR. Conservancies differ in their institutional arrangements; however, the common model found in the Mara occurs on privatised land and illustrates ‘group conservancies’, where groups of landowners have partnered with tourism operators to form a conservancy. The conservancy is primarily set aside for wildlife and tourism but with some controlled livestock grazing allowed. The partnerships involve individual lease agreements between the tourism operators and landowners, the most widespread of which involves landowners receiving fixed monthly land lease payments from the tourism operators, independent of the number of tourists visiting the conservancy (Bedelian 2014). The tourism operators also manage the conservancy and put up high-end tourism camps. Payments are made monthly straight to the bank accounts of individual landowners. In return, landowners must agree to vacate their land, remove settlements and livestock and refrain from any other land use within the conservancy. From an initial two conservancies in 2006, the conservancy model was quickly replicated in other areas of the Mara and by 2012 had increased to eight conservancies adjacent to the MMNR (Figure 1). In December 2015, there were 17 conservancies reported in the wider Narok County (Reid, RS, D Kaelo, KA Galvin, and R Harmon: Pastoral wildlife conservancies in Kenya: A bottom-up revolution in conservation, balancing livelihoods and conservation?, unpublished).

Most conservancies also set up a trust to channel donor funding to the conservancy and run community welfare projects. The trusts support schools and health centres, establish water points and other infrastructure and have microfinance and capacity-building programmes. They have set up and work with a number of women and school groups - groups recognised as left out of conservancies (Courtney 2009), since the land-titling process has meant only males are landowners and conservancy members.

This paper concentrates on the conservancies and households located in the former Koyiaki Group Ranch. Koyiaki lies on the northern border of the MMNR and supports an overspill of numerous wildlife from the MMNR in the wet season (Broten and Said 1995; Ogutu et al. 2008b; Bhola et al. 2012). Koyiaki is now fully privatised, and land was subdivided and allocated to group ranch members in a number of stages over a period of 25 years, beginning in 1984 (Thompson et al. 2009) and ending in 2009 when the final land blocks were subdivided. Koyiaki was one of the first group ranches to experiment with tourism revenue dispersal initiatives in the 1990s (Thompson and Homewood 2002) and also saw the emergence and rapid replication of conservancies from 2006^1^ (Bedelian 2014). There are now three conservancies in Koyiaki: Olare Motorogi Conservancy^2^, Naboisho Conservancy and the Mara North Conservancy. This paper focuses on these three conservancies.

## Methods

This paper draws on different sources of quantitative and qualitative data. Primary field data were collected in the Mara during 2010. A household questionnaire was administered to 258 household residents within Koyiaki Group Ranch. The main aim of the questionnaire was to compare livelihood activities and income levels, land ownership and grazing strategies among households. Individual study households (*n* = 258) were picked up using simple random sampling from a list of all resident households within Koyiaki Group Ranch (*n* = 1,825), drawn up with the help of a number of local informants. A household was defined as an *olmarei* (usually made up of a male household head, his wives, children and other dependents) - a common unit of analysis in previous household surveys among the Maasai in Kenya and Tanzania (BurnSilver and Mwangi 2007; Coast, E. 2000. Maasai demography. PhD Thesis, University of London, Unpublished; Thompson and Homewood 2002) and the most locally meaningful and representative unit of analysis in the Maasai context. The questionnaire was piloted before use with 15 households and translated into Maa to ensure questions were clear and well understood by participants (see Additional file 1 for the English version).

Approximately 30 semi-structured interviews were carried out with community members (CIs), including conservancy members and non-members, and men and women, to gather information on how people perceived conservancies contributed to their livelihoods. Approximately 30 interviews were carried out with key informants (KIIs), such as conservancy managers, committee members and tourism operators, to understand how conservancies operate and are managed. These interviews were more flexible than the questionnaire survey and were based on a list of prepared questions but were free to explore new topics as they arose.

Secondary sources of data include long-term livestock trend data collected through aerial surveys by the Directorate of Resource Surveys and Remote Sensing (DRSRS) from 1977 to 2014. These data were used to map the distribution and density of livestock species over time and in relation to conservancy boundaries.

### Livelihood activities and income levels

The Maasai have diversified into a number of other livelihood activities. Using the questionnaire, we asked each household what livelihood activities they were involved in during the year preceding the survey and the income they had received from each. We grouped activities into the following categories: conservancies, livestock production, cultivation and off-farm activities (tourism- and non-tourism-related), and calculated the mean annual household income from each activity (Table 1).

**Table 1:** Annual income accruing to households from the different livelihood activities

Livelihood activity	Made up of	Notes
Conservancies	Annual income received through monthly payments from one or more conservancy.	Some households were members of up to three different conservancies.
Livestock production	Gross revenue from livestock sold, value of livestock slaughtered, livestock gifts received and milk sold.	Sales of other livestock products, such as hides and skins, were sporadic and not captured here. The value of milk consumption was also not captured because we were unable to estimate it reliably from questionnaire data. Leaving out these products underestimates the value of livestock production, e.g., in Kenya, milk provides three quarters of the total gross value of livestock’s contribution to the agriculture sector, whereas hides and skins represent 4.3% of total livestock output (Behnke and Muthami 2011).
	In addition, data were collected on the composition and number of livestock owned by the household and transformed into Tropical Livestock Units (TLU)^a^.	
Cultivation	Income from crops sold and value of crops consumed.	Calculations included those households with a failed harvest but excluded those that had not yet harvested.
Off-farm activities:	Income from any other livelihood activity, including:	Many of the activities unrelated to tourism will be indirectly related to the increased flow of people coming to the Mara as a result of tourism.
Tourism-related sources	Jobs in tourism, income from curio and craft sales, rent fees from campsite or lodges.	
Non-tourism-related sources	Livestock trading, jobs such as teachers, health workers, income from a transport or vending shop business.	

^a^TLU take into account a range of livestock types and sizes in a standardised manner where 1 TLU = 250 kg. In this study, 1 cow = 0.72 TLU; 1 goat or 1 sheep = 0.17 TLU (Grandin 1988; ILCA 1981)

We calculated total annual household income for each household as the gross aggregate household income from all sources. Mean incomes per household per year and per adult equivalent (AU)^3^ per day were calculated for comparison among the different livelihood activities. We included only those households involved in an activity so as to make it possible to compare the real returns from each activity. However, we used mean annual incomes across all households in the sample (*n* = 258) to investigate the proportion of total household income the different livelihood activities contributed. Finally, we compared the relative contribution of different livelihood activities for conservancy member versus non-member households. To investigate community members’ perceptions of the importance of conservancies relative to other livelihood activities, we asked them to rank the three livelihood activities they perceived as the most important for their overall household welfare, using a guided list of common activities elicited through pre-testing.

### Conservancies and livestock grazing

To understand how conservancies affect livestock grazing, both inside and outside conservancies, we asked key informants about the grazing rules within conservancies and how grazing was managed. We also asked community members about their views on the way conservancies interacted with livestock grazing and how this affected their livelihoods. Discussions focused on the potential costs of conservancies for livestock grazing, such as in terms of lost grazing space or grazing fines, but also some of the benefits of conservancies for livestock, for example as important drought refuges.

To understand how conservancies were valued for grazing, we asked landowners to rank the parcels of land they owned in terms of key livestock-grazing attributes: (1) quality of grass, (2) quantity of grass, (3) proximity to salt licks, (4) access to water and (5) the tourism potential of the land. Chi-squared and *t* tests were used to compare conservancy and non-conservancy land. To investigate the importance of conservancies as livestock-grazing areas, we asked each household if they grazed their livestock in a conservancy in Koyiaki and, if so, how often. We also asked households if they used the MMNR for grazing to explore the importance of the MMNR for livestock grazing for pastoralists living adjacent to the MMNR. A number of quotes are used to discuss key issues that arose and highlight views of different members of society, for example men and women and conservancy members and non-members.

### Livestock trends

We analysed livestock count data collected through aerial surveys by DRSRS from 1977 to 2014, to investigate trends. Livestock were separated into cattle and shoats (sheep and goats combined). First, we analysed trends in cattle and shoats for the Mara ecosystem for the 1977 to 2014 period. A total of 60 aerial surveys were conducted from 1977 to 2014 (Additional file 2: Table S1). Of these, 30 surveys were conducted in the wet season months and the other 30 in the dry season months. There were thus sufficient surveys in each season to enable meaningful modelling of trends for each season from the time series of aerial surveys. We did not convert livestock numbers to Tropical Livestock Unit (TLU) because we wanted to examine trends in numbers of the different common species of livestock, primarily cattle, sheep and goats.

Then we looked at spatial and temporal trends in livestock in Koyiaki Group Ranch between 1977 and 2014 and compared livestock density and composition between areas inside and outside conservancies. We delineated areas in Koyiaki as either inside or outside a conservancy in ArcMap 10. Trends in cattle and shoat density are shown for inside and outside conservancies over this time period.

**Figure 2:**
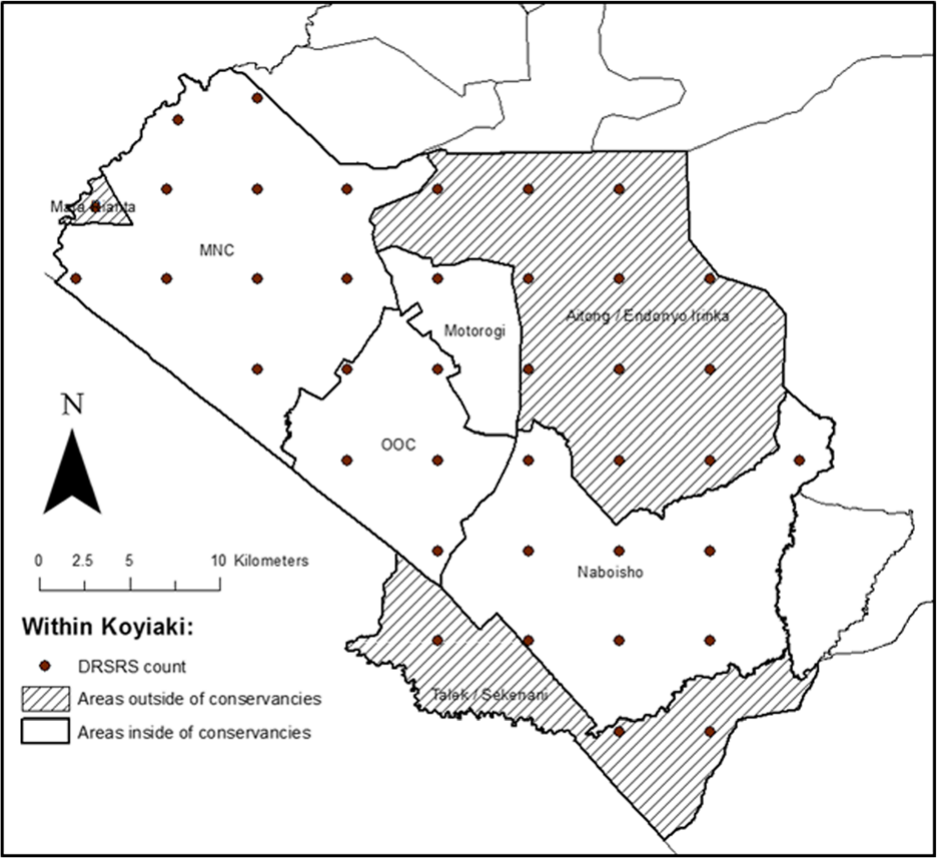
DRSRS 5 × 5 km^2^ sampling units inside and outside conservancies in Koyiaki Group Ranch

Livestock and wildlife trends in the Mara are well documented, with many analyses using the DRSRS aerial survey data (Bhola et al. 2012; Broten and Said 1995; Ogutu et al. 2011; Ottichilo et al. 2000). This analysis is the first attempt to look at livestock trends directly in relation to newly formed conservancy areas in Koyiaki. DRSRS population density estimates are based on transects subdivided into 5 × 5 km^2^ sampling units, each of which was identified as falling inside or outside a conservancy in Koyiaki (Figure 2). Norton-Griffiths (1978) and Ogutu et al. (2011) give further details of the method used to count animals and estimate animal population size and its standard error.

## Results

### Livelihood activities and income levels

Household socio-demographic characteristics of the final 258 households sampled in the questionnaire survey are shown in Table 2. The Maasai in Koyiaki diversify into a number of livelihood activities. Table 3 shows the number of households involved in different activities in the year preceding the household survey and the mean annual household income earned from each of these. The mean total annual household income was $4,334^4^ for the year 2009 to 2010. The distribution of income among all households was highly skewed, with the mean heavily influenced by a few wealthy cases. The median of $3,048 is hence a better representation of household income.

**Table 2:** Socio-demographic characteristics of the household questionnaire sample (*n* = 258)

	Min	Max	Mean	SD	Median
Gender of household head	13 female; 245 male				
Age of household head (years)	20	80	37.68	13.81	34
Education of household head (years)	0	14	3.01	4.78	0
Household size	1	24	9.04	4.78	8
Percentage of children 5 to 16 years in school	0	100	74.5	30.50	84.5

**Table 3:** Mean incomes from the different livelihood activities and mean total household income

Livelihood activity	HHs involved	% HHs	Per HH/year US$ (SE)	Per AU/day US$ (SE)	Median US$
Conservancies (*n* = 258)	133	52	1,135 (71.7)	0.41 (0.029)	963
Livestock production (*n* = 248)	241	97	2,504 (194.1)	0.91 (0.060)	1,510
Crop production (*n* = 258)	29	11	334 (88.0)	0.19 (0.054)	193
Off-farm tourism (*n* = 258)	158	61	1,081 (87.3)	0.49 (0.038)	816
Off-farm non-tourism (*n* = 258)	130	50	1,185 (126.0)	0.46 (0.045)	724
Total income	248	100	4,334 (244.3)	1.70 (0.076)	3,048

Note: Mean incomes are shown per household (*HH*) per year and per AU per day. Only includes those HH involved in the activity (standard errors shown in parenthesis)

**Figure 3:**
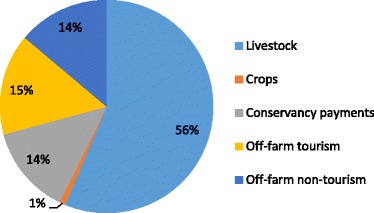
Contribution of different livelihood activities to total annual household income (%)

Figure 3 shows the contribution of different livelihood activities to total annual household income for all households. Conservancy payments contributed 14% of total annual income. Livestock production was the most important livelihood activity to Koyiaki households, contributing 56% of total annual income. Off-farm activities contributed 29%, split almost evenly between activities related (15%) or unrelated (14%) to tourism. Cultivation was negligible at 1%. We break down and explain each of these activities further below.

### Conservancy participation and income

From the survey of 258 randomly sampled households in Koyiaki, 52% (*n* = 133) received conservancy income through payments from at least one conservancy. The remaining 48% (*n* = 125) had no member who participated in a conservancy. In only 3% (*n* = 4) of the households was a woman a member of a conservancy. The mean annual income from conservancies for the 133 conservancy members, for the year preceding the survey, 2009 to 2010, was $1,135. In March 2010, Naboisho Conservancy was set up and began to distribute payments to its members. When this conservancy is taken into account, mean annual income from conservancies rose by 26% to $1,434, relative to the income expected for 2010 to 2011, the year following the survey.

### Livestock production

All households owned some livestock. The mean number of livestock (TLU) owned per household was 65; however, this varied considerably between households, from 3 to 390 TLU (Table 4). The number of TLU per AU also varied greatly, from 0.6 to 40, with a mean of 9 TLU/AU. Thirty-three percent of the households owned less than 5 TLU/AU - the lowest estimate of the threshold value required to support a purely pastoral lifestyle as estimated from a range of studies exploring numbers of livestock per capita (see Lamprey and Reid 2004 for a synthesis of studies and range of estimates).

**Table 4:** Number of livestock owned and household annual incomes (US$) from different livestock production activities

Livestock	% of HH	Mean^a^	Min	Max	SD	Median
Livestock owned (TLU)	100	64.8	3.0	389.2	62.9	45.3
TLU per AU	100	9.0	0.6	40.0	6.7	7.7
Value of livestock sold (*n* = 249)	92	2,276	18	21,313	2,923	1,199
Value of livestock consumed (*n* = 249)	73	182	11	1,198	158	143
Value of milk sold (*n* = 257)	12	280	19	1,755	356	141
Total livestock production (*n* = 248)^b^	97	2,504	21	22,073	3,014	1,510

^a^Only includes those households involved in a particular activity

^b^Total livestock production = livestock consumption + livestock sales + livestock gifts received + milk sales

Most households (92%) reported selling livestock, and the income from this (cattle, sheep and goats) brought in a mean cash income of $2,276 per household annually. Milk sales were not common: only 12% of households sold milk in the year 2009 to 2010, bringing in a mean annual income of $280. The mean annual gross value of livestock production was $2,504, with a median of $1,510, showing that a small number of wealthy households skew the mean up. This is a minimum estimate since it leaves out the value of milk consumption. Including the total value of milk could potentially put estimates from livestock 300% higher than are currently found here (Behnke and Muthami 2011).

### Cultivation

Cultivation made a negligible contribution (1%) to total annual household income. Few households (13%, *n* = 34) in Koyiaki cultivate, with most doing it for subsistence. Households cultivating were located along the northern edges close to Aitong or were cultivating outside of Koyiaki on neighbouring group ranches or further away in Trans Mara District, Mau and Narok, where there is higher potential for cultivation. Further south near the MMNR, there was no cultivation, except for patches in Sekenani and Nkoilale. No cultivation was reported in any conservancy in Koyiaki.

### Off-farm activities

The majority of households (87%) were involved in at least one off-farm activity, with a mean of 1.8 activities (Table 5). The mean annual off-farm income was $1,444 when including all sources, and off-farm activities contributed 29% of total household income. Activities related to tourism brought in a similar level of income to the household compared with activities unrelated to tourism.

**Table 5:** Households involved in off-farm activities and household annual income (US$) from off-farm tourism and non-tourism sources

Off-farm activities	% of HH	Mean^a^	Min	Max	SD	Median
No. of activities	87	1.8	1.0	5.0	1.0	1.0
Total off-farm income	87	1,444	4	10,409	1,560	956
Off-farm tourism income	61	1,081	47	7,500	1,097	816
Off-farm non-tourism income	50	1,185	4	7,904	1,437	724

^a^Only includes those households involved in a particular activity

### Conservancy members versus non-member households

**Figure 4:**
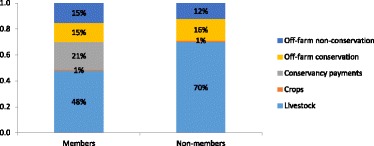
Proportion of gross annual household income from different activities, disaggregated into conservancy members (*n* = 127) and non-member (*n* = 121) households

When disaggregating households into conservancy member and non-member households, we find conservancy payments contribute 21% of the total annual income of member households (Figure 4). When accounting for Naboisho Conservancy, we expected the income for the year following the survey (2010 to 2011) to increase from 21% to 27%. The off-farm activity and cultivation contributions for member and non-member households are roughly similar.

However, livestock contribute a much larger portion of income for conservancy non-member (70%) than for member (48%) households. Thus, conservancy payments, although valuable to those households that receive them, are of limited value to the wider community, since almost half of households do not directly receive them. For these households, livestock remain far more important and make up the extra contribution; the other activities remain comparable across member and non-member households.

### Perceived importance of conservancies

**Figure 5:**
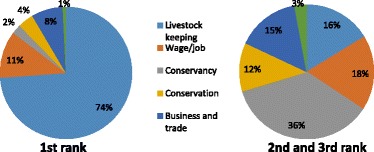
Perception of the importance of different livelihood activities to household welfare - conservancy members only (*n* = 131)

Conservancies were ranked as the household’s primary livelihood activity for welfare by only 2% of members, compared with livestock keeping at 74% (Figure 5). However, conservancies were consistently ranked as the most important second or third livelihood activity by those involved. Though members do not perceive involvement in conservancies as their main livelihood activity, which remains overwhelmingly livestock based, they do consider it an important supplement.

### Conservancies and livestock grazing

Conservancies influence grazing strategies in a number of ways. All conservancies have controlled grazing plans that set rules for within their boundaries. These usually restrict the number of livestock allowed into the conservancy and the areas where and the periods in which they can graze. Grazing plans vary by conservancy, but livestock grazing is generally restricted in areas surrounding tourist camps, to maintain higher quantities of grass and avoid livestock being visible to, or heard by, guests. Periodic grazing is allowed in areas farther away. Conservancies usually allow more flexible grazing during the low tourism season when some camps may be closed or during severe droughts. Some conservancies organise their grazing using a single but large grazing herd, opening up different parts of the conservancy in succession for grazing. Usually, cattle herds neighbouring the conservancy are allowed access on a rotational basis, as different parts of the conservancy are open for grazing at different times. Sheep and goats, which tend to graze close to their *bomas*, are not allowed access. Grazing is usually permitted only during the day for ease of monitoring and to avoid conflict with predators. Grazing rules are monitored and enforced by conservancy rangers, and herd owners are fined if herds are caught grazing in the conservancy outside of the specified times and places. Herds are driven out of the conservancy to the ranger post or gate and impounded until the fine is paid. In some cases, herders or livestock owners caught grazing illegally in the conservancies have been imprisoned or given community service (KII 32; Naboisho 2013). The commonest reason given for the strict grazing restrictions was the fact that tourism investors do not want cattle in the conservancies (KII 14, 18, 32). Where conservancies initially completely outlawed livestock grazing, some persuasion was required to convince the tourism investors to allow it to some level (KII 14). Although grazing restrictions applied to both conservancy members and non-members, there was discussion that when conservancies do allow livestock grazing, conservancy members’ livestock received preferential access (KII 16, 26; CI8, 17, 19, 29).

Conservancies are marketed and portrayed as exclusive, low-density tourism destinations that offer a private and authentic safari experience. For this reason, tourists do not expect or want to see cattle in the conservancies, and this is the argument the tourism investors gave (KII 32). Hence, grazing is carefully monitored within conservancies to avoid livestock or herders being seen by tourists. When cattle are allowed in, the herders must wear their traditional Maasai *shukas*. This argument generally takes precedence over any threat that livestock grazing might cause to the environment. In fact, the benefits of cattle grazing in maintaining a rich assembly of wildlife in these areas are well recognised by conservancy managers (KII 14, 19, 32) and documented by several studies (Augustine et al. 2011; Muchiru et al. 2008).

### Livestock-grazing conflicts

The restriction of grazing and reduction of space for livestock grazing in conservancies has been a contentious issue within the community, creating much conflict. Livestock owners complained that conservancies had seriously reduced the grazing areas available to them and prevented access to areas they once relied on. Moreover, during dry times, this issue has become accentuated in the search for forage for livestock. Thus, many viewed the livestock-grazing restrictions negatively, as imposing a big cost on their livelihoods:

*‘The worst rule is the one about grazing because you see the other issues like preserving the forest and stones are not bad. But it’s completely not good when they say the livestock should not graze up to a certain distance.’*

Group of men, members and non-members, community interview 10.

*‘I see it as a cost if the livestock are not allowed to graze in the conservancy because they will die during the drought season.’*

Woman, son is a member, community interview 11.

There was concern that the conservancies prevented access to important grazing resources that fall within their boundaries, such as salt licks, water sources and the *olkeri* (small grazing areas set aside for sick, old and young livestock):

*‘The bad thing about the olkeri is that we are not allowed to graze in it, whether you are a member of the conservancy or a nonmember.’*

Group of women, husbands are members, community interview 24.

*‘You might find that there are certain places which have water, and you are not allowed to go there. Do you think the cows can feed on grass without drinking water? You might also find there are some places with a salt lick, which they don’t allow. But can the cows enjoy the salt lick without eating grass?’*

Senior elder, member of Olare Orok Conservancy (OOC), key informant interview 29.

Women especially commented on the cost of grazing restrictions, since their livelihoods depend predominantly on livestock. They explained that the grazing restrictions might cause their livestock to die and this would mean they would not have food since they do not have anything else to depend on:

*W1: ‘We think it is a cost because our livelihood depends on the livestock only and if there are no livestock we have nothing to depend on.’*

*W2: ‘I see that life is starting to change as the number of your livestock will not be the same and the Maasai livelihood only depends on the livestock. If you don’t have livestock you don’t have food.’*

Group of women, husbands are members, community interview 12.

*‘The bad thing that we have seen about the conservancy is that they have refused us to go to the places where we have been grazing our cows, and they are what we depend on. So when they are refused to go to those places they have been grazing, they will die. And that will be difficult for us since what else will we be doing since our cows are what we depend on?’*

Group of women, husbands are members, community interview 25.

One point frequently made was that the payment received from the conservancy was not enough to compensate for livestock not being allowed to graze in the conservancy.

Commonly, comparisons were made between the size of the conservancy payment and the cost of livestock, as many saw the payment as low and not worth the cost of livestock that might die as a result of drought:

*‘When I receive that little money it’s a benefit, but there is not much benefit as that very little money is not worth the price of all of my cattle when they are dead due to the drought.’*

Senior elder, member of Mara North Conservancy (MNC), community interview 18.

Many thought of the payment as enough to cover some basic needs of the family, such as food and clothes, and to help pay for school fees or veterinary costs, but did not consider it an important part of their income. Very few reported using the money to actually buy livestock:

*‘I use the money to pay school fees, buy food, but it’s not enough to buy even a sheep …there should be free grazing since the lease fee is not worth the value of a steer.’*

Senior elder, member of OOC, community interview 14.

*W1: ‘I see it as very little because even when you sell your cow the amount that you receive from it you can buy more with than that which you receive from the conservancy.’*

*W2: ‘I just use it to buy foodstuff and then it is finished.’*

Group of women, husbands are members, community interview 12.

In these examples, the payment is compared with the value of livestock, with respondents feeling it fell short. This was a common comparison made by both men and women.

Although it is likely that the payment was enough to buy a cow in some cases, and certainly a sheep, many perceived it as too low for this. In these cases, the restriction on livestock grazing in the conservancy influences how people view the conservancy and its payments, which they believe are not enough to compensate for not grazing in the conservancy.

Women often tended to view the payment as small, since they do not have direct access to it. The payment is usually sent to their husband’s bank account, and they may or may not receive a portion:

*W1: ‘We use the little we get, but it’s not much that we can say it will provide for all our family needs.’*

*W2: ‘It’s only enough for the needs of the husband unless he decides to buy some food stuffs, since he will not give you any portion of it.’*

*Q: ‘If the women were the ones that received the money what would you do with it?’*

*W2: ‘We will do many things with it such as buying clothes and food, and paying to educate our children.’*

*W3: ‘And if some remains you can also buy a cow.’*

*W2: ‘But I don’t think it is enough to buy food and also a cow.’*

Group of women, husbands are members, community interview 25.

Others found the payment useful as it helped pay for school fees or in buying certain items:

*‘I am happy because I don’t sell my livestock anymore and I also use the money to pay for school fees for the children … I use the money for the education of my children.’*

Woman, son is a member, community interview 11.

*‘I can use the money to build a house like this one, which can be used for rent by other people (i.e. urban rental). I can also use it to take care of my livestock and family.’*

Elder, member of Naboisho Conservancy, community interview 9.

In these examples, although these respondents do not mention that they use the conservancy payment to buy livestock, they say they use it to provide for their livestock as well as their family. The payment also helps protect them from having to sell their livestock for cash needs, so it therefore provides them with a useful and regular source of cash. Although few reported spending their conservancy payments on fines received because of grazing in the conservancy, fines were perceived as a big cost for many people interviewed. Many conservancy members commented that what they had to pay for a fine was a similar sum to that received per month from the conservancy. This was made worse by the fact that they were being caught and fined for grazing on their own land:

*‘There is too much grass there, which the livestock are not allowed to feed on. It is also bad because we are caught and fined and this (land) is our property.’*

Senior elder, member of OOC, community interview 14.

*M1: ‘It is a cost if you are caught grazing your livestock on your land, and you have to sell the livestock to pay the fine.’*

*M2: ‘The bad thing that I can tell you now about the conservancy is when I am caught (grazing) on my own land, I am forced to sell my livestock, as they need me to pay KES 10,000, and that is the same amount I am paid by the conservancy.’*

Group of men, members and non-members, community interview 10.

*‘According to how I see it, the bad thing is the way people are being fined, because they can even fine somebody who doesn’t have any other job to depend on, and this will force him to sell his cows. I don’t see this as good at all.’*

Junior elder, non-member, community interview 27.

In these examples, people talked about having to sell their livestock to pay the fine, which they were obviously unhappy about. Fines tend to increase during the dry season (Bedelian 2014), as the ability of herds to find available forage decreases and conservancies come under considerable pressure from livestock grazing (see below).

### Livestock-grazing benefit

As well as conservancies being viewed as detrimental to livestock owing to grazing restrictions and fines, livestock grazing within conservancies was also seen as a benefit to livestock. Many spoke about conservancies having good livestock grazing, and when they did get to graze in the conservancy, it was a benefit. An important aspect of conservancies for livestock grazing was that the conservancy preserves the grass to be used during drought (CI 13, 23). Since livestock are not allowed to regularly graze in conservancies, this retains grass for use when needed, such as in the dry season or drought time. Thus, the conservancy acts as a grass bank or grazing reserve:

*‘Yes it helps the community by preserving grass, and when it becomes the dry season, we are allowed to graze, and that grass will last us some time.’*

Elder, non-member, community interview 13.

*‘We do like the way they are managing the grass because for those people who live nearby, they will get a chance (to graze inside).’*

Junior elder, non-member, community interview 27.

*‘Because nowadays we have plenty of grass because we don’t graze just anywhere. So during the drought season there is plenty of grass, and they can allow us to graze also there.’*

Elder, member of MNC, community interview 20.

Thus, by preventing widespread livestock grazing, conservancies retain grass, which, when accessed, is a big benefit. This was seen as particularly important during drought times. Conservancies were perceived as valuable pieces of land for livestock grazing. Households valued conservancy land significantly higher than non-conservancy land in terms of all four key livestock-grazing attributes: quality, quantity, water, salt lick and tourism (Table 6). When all five attributes were combined into a total score, conservancy land was rated significantly higher than non-conservancy land (*t* = 9.826, df = 365, *p* < 0.001). Conservancies are thus considered important areas for livestock and to have higher value than non-conservancy areas.

**Table 6:** Chi-squared tests for significant difference between how respondents valued conservancy (*n* = 200) vs. non-conservancy (*n* = 167) land

	Value *χ* ^2^	df	Sig
Quality of grass	12.7	3	0.005
Quantity of grass	29.1	4	0.000
Water	11.4	4	0.022
Salt lick	53.8	4	0.000
Tourism	111.9	4	0.000

### Reported use of conservancies and the MMNR for grazing

**Figure 6:**
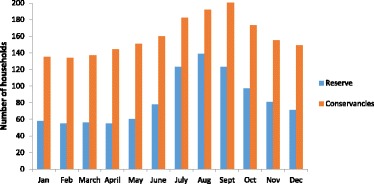
Monthly calendar of the use of the MMNR and conservancies (Olare Motorogi Conservancy, Mara North Conservancy and Naboisho Conservancy combined) by livestock herders in Koyiaki during 2009 to 2010 (*n* = 257)

Despite the grazing restrictions, many people still use the conservancies for grazing, both legally and illegally. Most households (87%) reported grazing inside conservancies, even outside of the agreed times, and about half of these households, consisting of both conservancy members and non-members, reported regularly grazing in conservancies. Conservancies were used for livestock grazing throughout the year, but this increased during the dry season (July to October) (Figure 6). During 2009, there was a very bad drought in the Mara, made worse by an influx of cattle from far off areas to seek grass (KII 14, 19). Many people from Koyiaki sought grazing in the conservancies, as the grass in areas outside became scarce. People reported grazing most heavily in OOC and MNC during this time, as these conservancies retained grass for longer than areas outside of conservancies and the drier Naboisho Conservancy to the east. Many people and their cattle also passed through OOC and MNC on their route out of the Mara as they journeyed west to Lolgorien in the neighbouring Trans Mara area, a higher area with more rainfall, where grazing conditions were more favourable. During this time, conservancy managers reported conservancies were under considerable pressure from livestock grazing (KII 14, 15). Wildlife were dying as a result of lack of forage, so conservancies closed their boundaries for livestock grazing.

Many cattle herds were removed from the conservancy and the herd owners fined (KII 18).

Thus, although drought periods are a time when there is much pressure from livestock owners to graze within conservancies, these are not necessarily periods when the grazing rules are relaxed. Much of this can also be explained by the fact that the dry season coincides with the high tourism season in the Mara and when the wildebeest migration arrives from the Serengeti. This thus creates conflicts between livestock herders and the conservancy during this critical period. Nevertheless, conservancies remain important livestock-grazing areas for many people. There were instances where people moved their settlements closer to the conservancy boundaries so livestock were able to better access grass within conservancies (KII 33).

The MMNR was also found to be an important livestock-grazing resource for herders. Although grazing in the MMNR is illegal, rangers in the past have turned a blind eye and tolerated grazing if only at night when there are no tourists inside the Reserve. Sixty-six percent of households reported using the MMNR for grazing, and most of these households (64%) reported grazing in the night-time only to avoid the risk of being fined by rangers. Similar to the conservancies, the heaviest livestock-grazing pressure in the MMNR was found during the dry season (Figure 6). Again, by coinciding with the peak tourism season in the MMNR, this creates conflicts between herders and the Reserve at this time.

### Livestock trends

**Figure 7:**
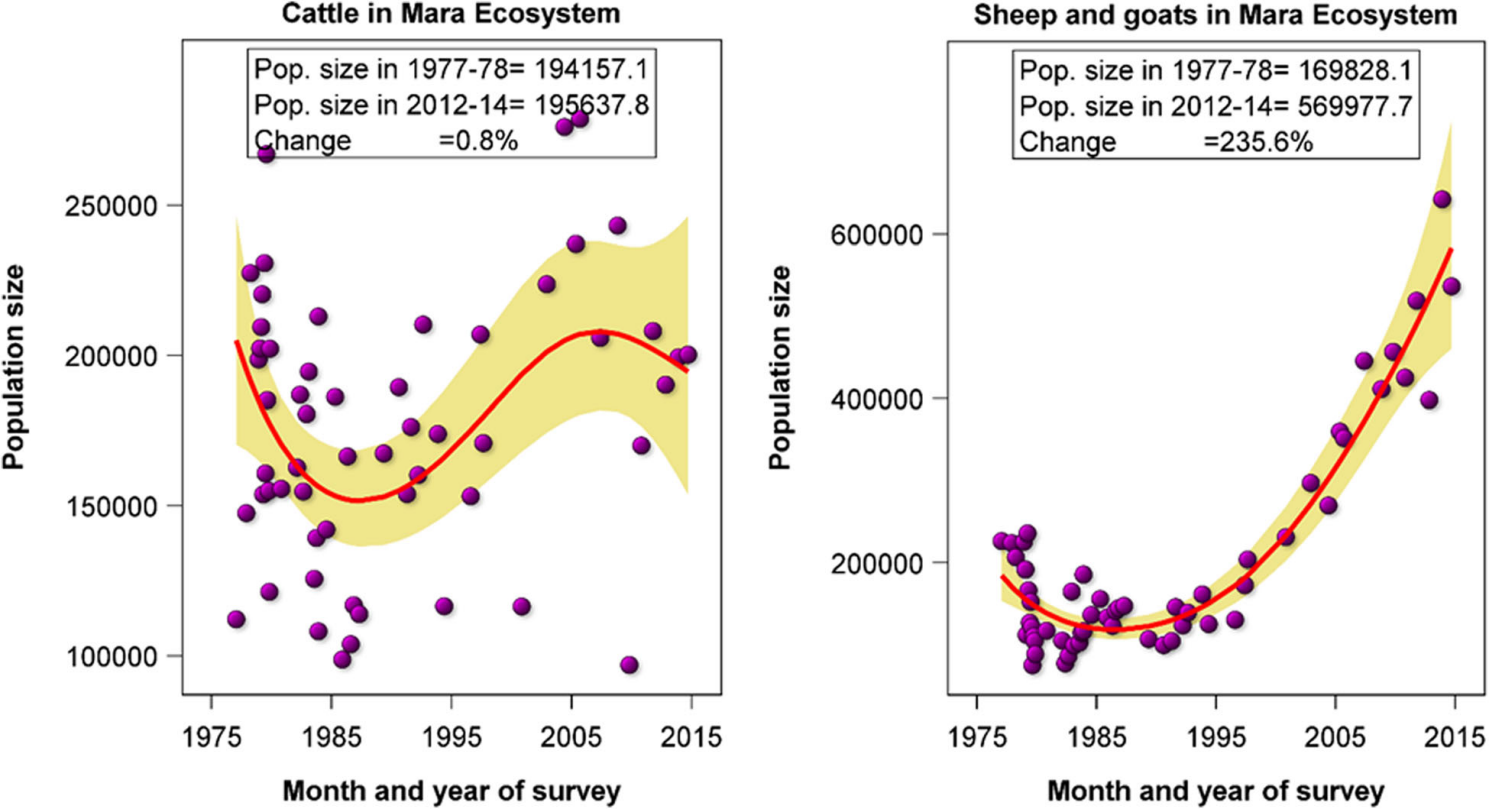
Trends in cattle and shoat populations in the Mara ecosystem from 1977 to 2014

Livestock in the Mara include cattle, shoats and, to a lesser extent, donkeys. Figure 7 shows trends in cattle and shoat populations in the Mara ecosystem from 1977 to 2014. The cattle numbers show strong inter-annual variability and large seasonal differences according to wet and dry season counts but with a very small overall upward trend evident between 1977 and 2014 (+0.8%). In comparison, the number of shoats shows a very large upward trend during 1977 to 2014 (+235.6%) but with less inter-annual and seasonal fluctuations than for cattle. These patterns reaffirm those of Ogutu et al. (2011), who found the number of shoats almost tripled, whereas cattle numbers varied widely but with an apparent overall increase outside and a marked and significant increase inside the MMNR. The accelerated increase in shoats from around 1995, but low overall increase in cattle, suggests a greater reliance on small stock in recent years. The data show there are now (2014) almost three times as many shoats in the Mara as there are cattle.

### Livestock trend analysis in relation to conservancies

**Figure 8:**
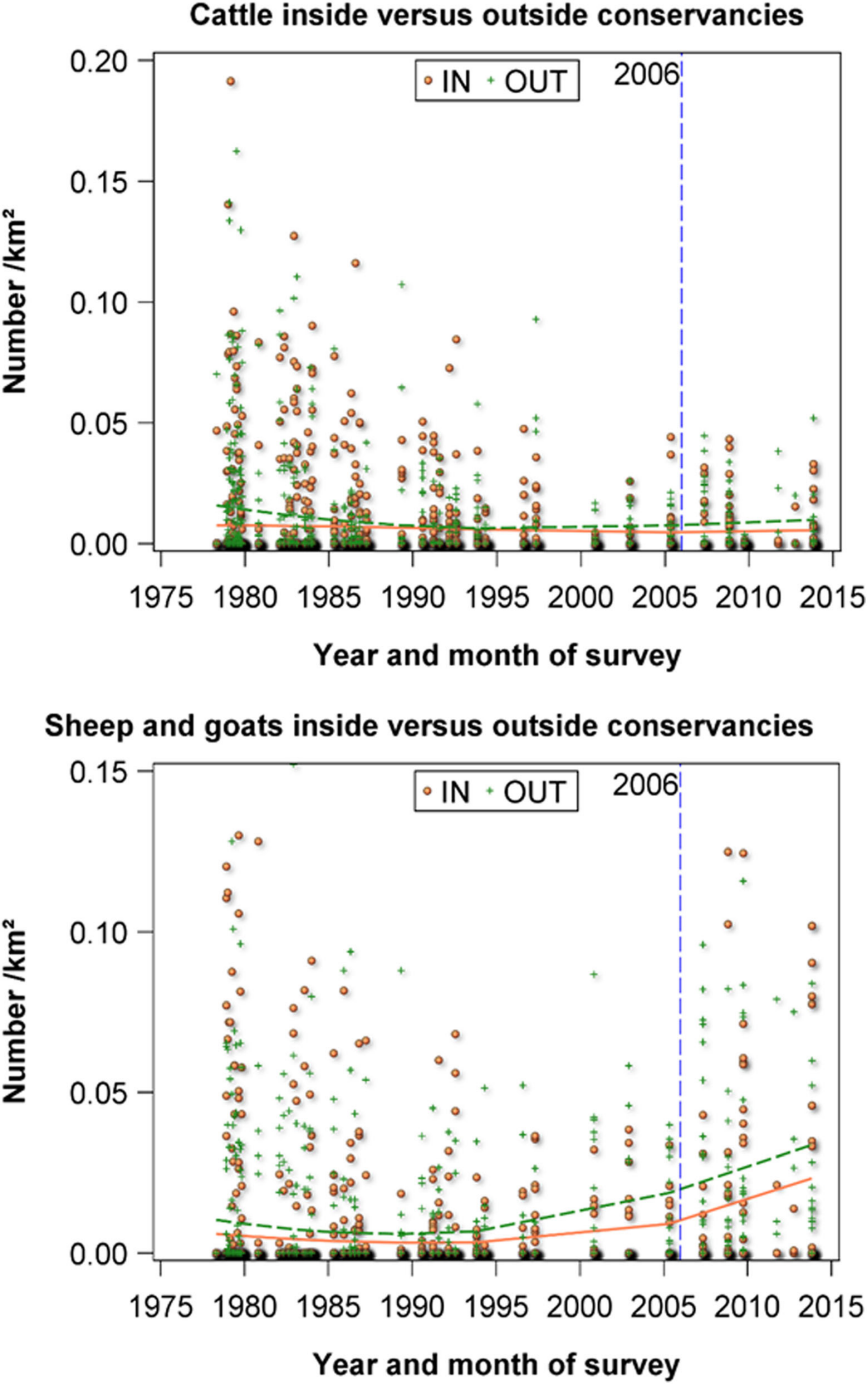
Trends in cattle and shoat density, inside and outside of conservancies, 1977 to 2014, Koyiaki Group Ranch. Figure note: *Coral circles* and *solid lines* denote inside conservancies and *forest green triangles* and *dashed trend lines* outside conservancies. The *dashed blue line* indicates when conservancies began to expand in number and size in 2006

So what do livestock count data show us about how conservancies are affecting the density and composition of livestock, inside and outside of conservancies? Cattle densities are highly variable and show little discernible change in density over time but with severe drops in density evident during well-known drought periods in 1999 to 2000 and 2009 (Figure 8 on cattle). After a crash in cattle numbers following the 1999 to 2000 drought, cattle numbers began increasing slowly. For most of the monitoring period, the density is lower inside than outside the conservancies. However, this increase appears to be accelerating in recent years and following the setting-up of conservancies in 2006. Although the density of shoats is equally highly variable, it shows a more obvious trend over time (Figure 8 on shoats), notably a large and sustained increase from 1996 to 2014. Similar to cattle, fewer shoats were found inside than outside conservancies, but this trend is much more clearly apparent for shoats.

Overall, livestock, including shoats, are still using the conservancies to quite a considerable extent. Therefore, despite conservancies trying to reduce livestock grazing, it is clear a great deal of grazing is still occurring inside them. Given the few years of available data since conservancies were set up, and the variability in livestock numbers, these trends should be interpreted cautiously.

## Discussion

### Livelihood trade-offs

Conservancies are an important livelihood activity for those who participate and can make a significant contribution to overall household income. During 2009 to 2010, conservancies contributed 21% of total household income for Koyiaki conservancy members. This was expected to increase to 27% the following year, as more conservancies became established.

Conservancy payments were also found to be an important source of cash income, helping households avoid selling their livestock for cash needs. Since payments are guaranteed and fixed every month, this provides a reliable all-year-round source of income and can protect households from having to sell their animals during times of stress. Conservancy payments thus play an important role in buffering against droughts and reducing risk during climatic shocks when other sources of income may decline. Similarly, Osano et al. (2013) found conservation payments became particularly important during drought in two sites (the Mara and Kitengela) in Kenya in 2009, when income from livestock declined.

However, the limitations placed on livestock mean conservancy payments come at the expense of livestock production. For livestock owners, there are thus opportunity costs and livelihood trade-offs attached to participating in conservancies. The opportunity costs of lost grazing are particularly pertinent given that livestock are overwhelmingly the most important activity for Koyiaki households; all households own livestock, livestock contribute the most to total household income (56%) and livestock are valued as the most important activity for household welfare. For those non-members who do not receive conservancy payments, the contribution of livestock is much higher - 70% versus 48% for those who receive payment - and these households depend on livestock more than any other activity. This is the case even though milk consumption and livestock products such as skins and hides are not included in total livestock production income, therefore undervaluing the contribution of livestock. Estimates of livestock contribution could be as much as 300% higher if milk production, which contributes nearly three quarters of the total gross value of livestock’s contribution to the agriculture sector, is taken into account (Behnke and Muthami 2011).

Since payments do not adequately compensate conservancy members for the restrictions they put on their other livelihood activities, members did not perceive conservancies as their main livelihood activity but as an important supplementary livelihood source. This is a common finding from research assessing tourism’s contribution to pastoral livelihoods; pastoralists rarely view tourism as a substitute for their usual livelihood activities, but rather as a possible way of supplementing them (DeLuca 2004; Homewood et al. 2009). The fact that people conceptualised the payment amount against the value of a cow tends to show they value livestock more.

Livestock thus remain central to the livelihoods of most rural Maasai and represent their core economic and cultural strategy (Homewood et al. 2009). There are multiple and flexible ways that livestock are integrated into Maasai livelihoods: as a wealth store, as an investment yielding growth in herd numbers, as a food source (milk) and as producing animals for sale. There is also the cultural and social value attached to owning livestock, outside of its economic value. In comparison with conservancy payments, which are banked by the individual landowner and thus not as unquestioningly redistributed or shared among the family, livestock offer benefits to the whole household in both direct and indirect ways. Livestock income is more easily distributed in cash and in-kind among various members of the family, including, for example, women, who typically accrue small but significant sums from sales of milk and hides. Even if not under their direct control or ownership, livestock are a source of subsistence, income and social status for women through control of livestock products (Njuki and Sanginga 2013; Talle 1988). Conservancy payments thus offer little comparative benefit and significant disadvantages to women, especially when considering the restrictions conservancies place on livestock. This can explain why people, and especially women, saw little value to the conservancy payments and valued them lower than livestock. Although women are included in conservancy co-benefits such as community training and capacity building, these activities do not compensate for the loss of livelihood activity because of the central contribution of livestock to family food systems, income and socio-cultural well-being.

Payments are also of limited value to the large portion of the community that are not conservancy members and so do not receive conservancy payments. In addition to women and landless households, who by virtue of not owning land are ineligible to join conservancies, there are a number of conservancy non-member households that live outside of conservancies. These non-members experience the cost of lost payment in return. They must also accommodate conservancy member livestock on their land.

Although cultivation potentially offers the highest returns on land use (Norton-Griffiths and Said 2010), this study found that few people cultivate and fewer people gain much from it. Previous research has shown little uptake of cultivation in Koyiaki, owing to increased occurrence of droughts, wildlife damage and competing interests with tourism (Thompson et al. 2009). Cultivation might be used more as a tenure strategy; cultivation peaked before land titling, and reduced thereafter once land was secured (ibid.).

### Synergies with pastoralism

As well as the trade-offs, conservancies were found to have important synergies with livestock keeping. Conservancies, since not as regularly accessed by livestock, retain a good quality and quantity of grass and are thus important grazing areas if accessible during drought times. People valued conservancy land significantly higher for livestock grazing than they did non-conservancy land and still grazed illegally inside conservancies despite the risk of being fined. Before conservancies were set up, these areas generally had fewer settlements and livestock, possibly due to a lack of permanent water, the presence of tsetse fly and often being situated further from towns (Bedelian 2014). Many conservancies also have direct and close access to the MMNR. These findings point to the favourable conditions for livestock, and also wildlife, especially as drought grazing areas.

Although conservancies restrict access to and use of pastoral grazing land, they also keep rangeland open by pooling individual parcels of land and keeping them free of fencing, cultivation and other land-use developments, thereby being consistent with mobile livestock keeping. The subdivision and individualisation of land cause land fragmentation, reduce access to grazing land and constrain livestock mobility (Galvin et al. 2008). Conservancies help maintain open rangelands by keeping land together that otherwise might have been further subdivided, fenced and sold. By offering monetary incentives, conservancies discourage landowners from subdividing or selling their land and thus can facilitate open rangelands and be beneficial to both pastoralism and wildlife.

Thus, this post-subdivision re-aggregation has allowed land to revert to something resembling its pre-subdivision state and helps maintain open rangelands in the Mara. Subdivision resulted in most people having parcels too small for their livestock, imposing limits on where they might graze. Conservancies can now maintain livestock mobility over large grazing areas, if and when allowed.

Nevertheless, this argument dismisses pastoralists’ ability to negotiate and maintain access through social networks, which allow reciprocal use and pasture sharing (BurnSilver and Mwangi 2007). In the neighbouring Kajiado County, BurnSilver and Mwangi describe how Maasai landowners re-aggregated their land parcels post-subdivision through pasture-sharing and swapping mechanisms to maintain access to resources. This is built on pre-existing cultural norms, such as herd redistribution and stock-sharing relationships. These arguments point to the mix of synergies and trade-offs involved in conservancies and pastoralism and also the possible positive potentials.

### Livestock trends

It is clear from the long-term trend data that shoats are rapidly increasing in number in the Mara, whereas cattle numbers show a much less marked upward trend. This evident switch to small stock is likely a response to widening variability in cattle numbers, which probably reflects their greater sensitivity to widening rainfall variability than shoats (Faye et al. 2012; Seo et al. 2009). Shoats have greater capacity to recover more rapidly from droughts. The switch to small stock can also be explained by the lower feed requirements and shorter gestation time of small stock and the important role they play in rebuilding herds when recovering from drought (McPeak and Little 2005). The switch to small stock is also a common strategy where mobility is increasingly curtailed (Dahl and Hjort 1976) and is expected to increase with climate warming (Seo and Mendelsohn 2008). The switch is also a trend mirrored in other pastoral areas (e.g. Ottichilo et al. 2000). The diversification of herds is thus an important strategy that pastoralists use to manage and cope with risk presented by a variable climate and to reduce their vulnerability to recurrent droughts.

Since conservancy-grazing restrictions limit grazing areas and curtail mobility, it is possible they are contributing to the greater reliance on shoats evident in the Mara. This hypothesis suggests that, since small stock require smaller grazing ranges and less forage, and so are better able to graze on the short grasses closer to the *boma*, they are preferred over cattle in a landscape with limited mobility. However, when serving as grass banks, conservancies may also increase mobility (or access to forage) when times are hardest and thus allow for possible synergistic relationship to exist between livestock and conservancies, in which conservancies may enhance long-term but controlled livestock mobility.

Analysis of trends in livestock density in relation to conservancies in the Mara shows it is not yet clear what the effects of conservancies are on livestock. Given the relatively recent implementation of conservancies, the mobile nature of livestock and the large variability in livestock numbers, a longer timeframe in which to observe any change might be needed. However, three main points emerge.

First, areas inside the conservancies have fewer livestock than those outside, and this was apparent even before conservancies were set up. These areas have generally had poorer access to water and market centres, some have been sites of previous tourism initiatives and some have suffered from the presence of tsetse, which are all likely to have been important contributing factors preventing heavy settlement and livestock grazing (Bedelian 2014).

Second, livestock appear to be increasing more rapidly outside than inside conservancies, and this is more visible for shoats than for cattle. However, it is as yet not possible to definitively ascribe this to conservancies.

Third, despite livestock-grazing restrictions, conservancies are still heavily grazed by livestock, including shoats. This is supported by data from livestock owners. Thus, conservancies are still important grazing areas for livestock and are regularly used either legally or illegally.

The influence of conservancies on livestock remains unclear. However, the reduction in space for livestock use owing to conservancies is beyond doubt. Aware of this, conservancies are encouraging their members to reduce their herds. By setting up controlled grazing plans with specified stocking densities, and by promoting and introducing improved or exotic breeds to members to mix with their traditional breeds, conservancies aim to reduce pressure on conservancy land by reducing livestock numbers. The idea is that improved breeds, when crossed with local breeds, can be more productive and can ultimately fetch a higher market price. The presumption is that this will then encourage landowners to own less livestock but of a higher quality relative to their traditional breeds.

Having fewer animals is expected to reduce the impact of livestock on the environment. However, although improved cattle breeds have higher market values, they have been found to be less resistant to droughts and floods than local breeds and require more veterinary and fodder inputs (Nkedianye et al. 2011). There is also no evidence that the adoption of more productive breeds necessarily translates into people keeping fewer animals (Marshall 2014). Conservancy cattle management plans are influenced by commercial ranching models, and many are based on experience and expert opinion from ranchers and land managers in other mixed livestock and wildlife areas in Kenya. This implies a change to many of the long-term traditional and customary practices of livestock grazing and management in the area.

The conservancies’ grazing restrictions have also likely put pressure on the MMNR as a livestock-grazing resource. The heavy use of the MMNR for livestock grazing and its importance during dry periods found here highlight the importance of the MMNR as a grazing resource, even though illegal. Although livestock have historically used the reserve during drought times, recent studies have shown an increasing use of the MMNR by cattle (Ogutu et al. 2011) and even pastoralists preferring to graze inside the MMNR rather than the conservancies to maintain their eligibility of payment by a conservancy (Butt 2011). Although most grazing in the MMNR reported here was during the night, there is a considerable recent surge in regular illegal livestock grazing in the MMNR during both the daytime and night-time (Joseph Ogutu, personal observation 2017). Also of concern is the unprecedented and widespread increase in the number of fences (Løvschal et al. 2017) and emergence of new sites of elevated settlement densities, in part linked to the reduction in livestock-grazing areas and redistribution of settlements following the establishment of conservancies. These points question the conservation effectiveness of conservancies, and conservancies will be less effective in promoting wildlife conservation if conservancy earnings are invested in purchasing more livestock or erecting fences, or if livestock numbers, fencing and illegal livestock incursions into the Mara Reserve are not effectively regulated.

### Trade-offs for climate-resilient livelihoods

This paper has pointed to some of the trade-offs involved in participating in conservancies.

Conservancies offer regular and reliable payments all year round and can be an important source of income when others, such as livestock incomes, dry up. Payments can thus buffer some of the variability in pastoral systems and risks arising as a result of droughts or climatic events. Conservancies also retain grass banks during the dry season and thus provide opportunities for pastoralists to access good-quality forage when they most need it. The establishment of water points through conservancy trusts has also helped pastoralists and their livestock access new water sources (Dickson Kaelo, personal communications). In these ways, conservancies can be viewed as an important drought-coping and risk mitigation strategy helping reduce pastoral vulnerability.

However, the restrictions placed on livestock in conservancies bring about opportunity costs and trade-offs. Mobility is one of the main coping strategies pastoralists use during droughts; thus, if conservancies restrict mobility, this affects pastoralists’ ability to remain flexible and be able to access variable resources. Also, if particularly important resources are removed, such as water, salt licks and the *olkeri*, this will have repercussions for the condition of livestock, which may be critical during drought periods or undermine recovery in their aftermath. Furthermore, conflict arises between livestock grazing and conservancies during drought times or the dry season as this coincides with the high tourism season, a time when both wildlife and tourists are in the Mara in higher numbers. Thus, conservancy effects on livestock might be quite mixed and will differ depending on the extent that livestock mobility is maintained and livestock grazing is integrated into conservancy management plans.

These trade-offs are all the more pertinent because livestock is the most important economic activity for the livelihoods of households studied here. Moreover, livestock represent a cultural dimension to pastoralism, beyond their economic value, which is harder to replace with tourism and conservation activities and goals that have a more Western value.

These trade-offs may also vary within the household and be different for women than men. Since women do not directly receive conservancy payments, this may heighten their exposure to climate change risks if they deviate from their resource-based livelihood activities. Women thus experience different livelihood trade-offs because of their different roles and gendered livelihood activities. Wangui (2014) shows how men and women construe climate change differently in pastoral areas in Kenya where gendered roles and responsibilities are still relatively well defined. Women feel the risks of climate change differently to men because of their gendered societal roles (ibid.). This may explain their assignment of greater value to livestock than conservancies and highlight the greater trade-offs they are exposed to. This stresses the importance of looking at conservancies for the whole family and not just the male-headed conservancy member.

Although pastoralism does have synergies with conservation and tourism, it is also important to consider the resilience and sustainability of these types of tourism schemes. Conservancies rely on a continuing tourism in the Mara, to be able to finance members’ payments. However, tourism is susceptible to concerns over political stability, economic downturns and violence, and there are many cases of tourism dropping because of such political and economic shocks. Climate change also has the potential to affect the tourism industry, such as through the large-scale destruction of infrastructure by floods as happened during the 1997 El Niño and the current flooding of national parks (Nakuru, Navaisha and Bogoria) containing Rift Valley Lakes. These insecurities make tourism a risky livelihood alternative to pastoralism.

Nevertheless, given the extent of change in these systems, and the fact that land subdivision and fragmentation are altering the way the system has traditionally functioned, people increasingly need to and do engage in new livelihood activities (Galvin 2009). Livestock numbers per capita are going down, also as a function of subdivision (Thornton et al. 2006), and there is some evidence that this is true of the Mara as well. This study found an average of 9 TLU per AU in 2010, down from 13 in 2004 and 15 in 1998 to 2000 found by Thompson et al. (2009). Furthermore, in this study, 33% of households owned less than the estimated threshold value of livestock per capita required to support a purely pastoral lifestyle.

Income from other sources and diversification are thus increasingly becoming an important activity and source of wealth (Homewood et al. 2009; Little 2012; McPeak et al. 2012). As land is subdivided, and individually owned, mobility is curtailed and people have to compensate for the potential loss of movement by engaging in new land uses and other ways of earning a living (Galvin 2009). Indeed, pastoralists are changing, diversifying and modernising. Other areas of Kenya show an ongoing intensification of livestock, through cross-breeding traditional breeds with large breeds of greater market value (BurnSilver and Mwangi 2007; Nkedianye et al. 2011). Many are investing in education, diversifying their incomes, taking up new opportunities and innovations and accessing and capitalising on new markets (Catley et al. 2012; Homewood et al. 2009). It is likely that adaptation to climate change will require more to pursue alternative livelihoods and some to move out of pastoralism altogether into other livelihoods (McPeak et al. 2012).

This paper draws on primary data collected in 2010, and as with the rapid evolution of conservancies in the Mara and wider Kenya (Reid, RS, D Kaelo, KA Galvin, and R Harmon: Pastoral wildlife conservancies in Kenya: A bottom-up revolution in conservation, balancing livelihoods and conservation?, unpublished), conservancy management plans and policies do also evolve. Conservancies are continually adapting and making changes to the way they operate. This needs to be acknowledged in any interpretation of the results. The paper also focuses on the three conservancies in Koyiaki, and the observations made here may not all be generalised to all the Mara conservancies or to conservancies elsewhere in Kenya, which come in many different forms and models.

## Conclusions

This paper has explored the opportunities and conflicts that emerge for climate-resilient pastoral livelihoods for landowners who participate in wildlife conservancies in the Mara, Kenya. Results show that, though offering stable payments (based on a stable tourism in the Mara), conservancies cause trade-offs as livestock and other livelihood activities are restricted. This reduces the ability to access resources, remain mobile and maintain resilience. Also, because the income received from conservancy payments is not more than that received from livestock production, conservancies do not adequately compensate landowners for the restrictions placed on their other livelihood activities. Moreover, since conservancy payments are limited to those owning land inside a conservancy, a large portion of the community do not receive conservancy payments but still experience the cost of lost livestock-grazing space. This includes women and other groups not allocated land during subdivision.

However, community members also recognised the benefits of conservancies for livestock grazing and pastoralism. Conservancies retain good quality and quantity of grass and are important livestock-grazing areas if accessible during drought times. Conservancies also pool land and prevent further subdivision and fragmentation. Thus, given the extent of land tenure changes in the Mara, conservancies and other similar schemes that maintain open rangelands could offer a potentially optimistic outlook for these areas, provided livestock are accommodated for. Conservancy effects may therefore be mixed and dependent on the policies and practices of individual conservancies and of the landowners’ continuing motivations to participate.

Conservancies are not fully integrative, and like other schemes in Maasailand (Homewood et al. 2012), they aim to replace livestock, rather than to fully integrate with livestock within the same landscape. Livestock support livelihoods and can contribute to protecting biodiversity; livestock landscapes thus need to be part of the conservation agenda. There is a need for better-thought-out integrative livestock-grazing plans, for better integration of pastoralism and tourism within and beyond conservancies. These need to acknowledge the risk management benefits associated with livestock, transmission of diseases between wildlife and livestock and the cultural and social values attached to livestock by the whole family. These need to be taken into account beyond any simple economic appraisal of conservancies or similar livelihood activity.

Pastoralists have always had traditional strategies to regulate the access and use of resources and to cope with climatic variability. These include regulations on how many herds access a particular grazing area or when they move to dry season areas or access important resources, such as salt or water, ensuring there is adequate remaining for others. Conservancies could do well to draw on and mimic such traditional grazing strategies, developing their livestock-grazing plans together with livestock keepers, including both conservancy members and non-members.

The Mara is a unique case study; it is the highest wildlife-earning site in Maasailand (Homewood et al. 2009), and its impressive wildlife abundance and diversity make it one of the top most visited tourist attractions in Kenya. Being at the top end of tourism revenue potential means conservancies are able to offer relatively large payments on a wide scale in the Mara. It is not certain that similar schemes in other areas would be able to offer pastoralists as much. However, conservancies are growing across Kenya and being widely adopted by local communities (Reid, RS, D Kaelo, KA Galvin, and R Harmon: Pastoral wildlife conservancies in Kenya: A bottom-up revolution in conservation, balancing livelihoods and conservation?, unpublished). Although they vary considerably in terms of their ownership and management arrangements, this Mara case study provides valuable lessons for what could potentially occur in other sites.

### Recommendations

- Carefully formulated livestock-grazing plans are needed to allow for better integration of, and space for, livestock within and outside of conservancies. These should recognise the need to conserve good-quality rangeland for livestock, similar to how the conservancies expand and conserve habitat for wildlife. This should occur through a participatory process, not just with conservancy members but also with women, herders and other non-members who reside next to a conservancy.
- It is important that grazing plans are holistic and encompass areas outside of conservancies. They should analyse their impact on the MMNR as well as focusing on land within the conservancy to avoid the problem of leakage and degradation to areas outside.
- An increased focus on conservancies as areas managed for livestock as well as their current focus on tourism and wildlife conservation is needed. This should involve the identification of critical areas and periods where conflict between livestock and tourism is likely to increase and will need mitigation with appropriate strategies.
- There is opportunity for better integration of livestock in conservancy marketing, so tourists are aware from the outset and expect to see livestock are integrated into conservancies.
- Better inclusion of non-conservancy members in conservancy operations is necessary. This includes in livestock-grazing plans but also in conservancy management and in the distribution of conservancy payments.
- Clear policy guidelines for the development of conservancies, adequate benefit sharing, participatory processes and sustainable land use are required.

## Additional files

Additional file 1: Koyiaki household questionnaire. (DOC 349 kb)

Additional file 2: Table S1. Table showing the dates and seasons of the 60 aerial surveys conducted in the Mara ecosystem between 1977 and 2014 by the Directorate of Resource Surveys and Remote Sensing (DRSRS). (DOCX 15 kb)

## Footnotes

1 The Olkinyei Conservancy in the adjacent Olkinyei Group Ranch was established one year earlier in 2005.

2 The Olare Orok Conservancy was established in 2006, and a year later, the Motorogi Conservancy was established as an extension to Olare Orok. These conservancies combined in 2012 to form the Olare Motorogi Conservancy.

3 AU is a system for expressing a group of people in terms of standard reference adult (RA) units, with respect to food or metabolic requirements. RA units were calculated according to the International Livestock Centre for Africa (ILCA) system whereby an adult male = 1RA; an adult female = 0.86RA; children 11 to 15 years = 0.96RA; 6 to 10 years = 0.85RA; and children 0 to 5 years = 0.52RA (ILCA 1981; Sellen 2003).

4 Exchange rate of $1 = 80 KES in 2010, as used in the rest of the paper.
