# Supplementary material for: Trade-offs for climate-resilient pastoral livelihoods in wildlife conservancies in the Mara ecosystem, Kenya
Source: Pastoralism. 2017 May 23;7(1):10. doi: 10.1186/s13570-017-0085-1 (PMC6991982; doi:10.1186/s13570-017-0085-1)
Supplement: Supplementary file 1 — Koyiaki household questionnaire. (DOC 349 kb) [file 13570_2017_85_MOESM1_ESM.doc]

**Koyiaki Household Questionnaire**

1. **Survey Information**

| 1. Date |  | | | |
| --- | --- | --- | --- | --- |
| 1. Town |  | | | |
| 1. Sub-area |  | | | |
| 1. GPS ID |  | | | |
| 1. GPS X   GPS Y |  | | | |
|  |  |  |  |  |
| 1. Interviewer/s |  | | | |
| 1. Start Time |  | | | |
| 1. End Time |  | | | |
| 1. **Background information** | | | | |
| 1. Name of respondent (household head) |  | | | |
| 1. Gender | M | | F | |
| 1. Marital status | Married | Single | Widowed | Divorced |
| 1. Have you ever held a leadership position?   If YES, what?  For what time period? | YES | | NO | |
|  | 1) 2) | | | |
|  | 1) 2) | | | |

1. **Please define your olmarei**

| No. | Relationship to household head | Age | Sex | Level of education reached or current schooling* | Occupation** | | Location:  Household (1) Elsewhere (2) *– (where?)* |
| --- | --- | --- | --- | --- | --- | --- | --- |
|  |  |  |  |  | 1st | 2^nd^ |  |
| 1 |  |  |  |  |  |  |  |
| 2 |  |  |  |  |  |  |  |
| 3 |  |  |  |  |  |  |  |
| 4 |  |  |  |  |  |  |  |
| 5 |  |  |  |  |  |  |  |
| 6 |  |  |  |  |  |  |  |
| 7 |  |  |  |  |  |  |  |
| 8 |  |  |  |  |  |  |  |
| 9 |  |  |  |  |  |  |  |
| 10 |  |  |  |  |  |  |  |
| 11 |  |  |  |  |  |  |  |
| 12 |  |  |  |  |  |  |  |
| 13 |  |  |  |  |  |  |  |
| 14 |  |  |  |  |  |  |  |
| 15 |  |  |  |  |  |  |  |
| 16 |  |  |  |  |  |  |  |
| 17 |  |  |  |  |  |  |  |
| 18 |  |  |  |  |  |  |  |
| 19 |  |  |  |  |  |  |  |
| 20 |  |  |  |  |  |  |  |
| 21 |  |  |  |  |  |  |  |
| 22 |  |  |  |  |  |  |  |
| 23 |  |  |  |  |  |  |  |
| 24 |  |  |  |  |  |  |  |

**Education- If currently schooling, circle the response level*

***Occupation- ask the respondent what occupation they spend the most time doing. The occupation doesn’t have to be related to income*

1. **Settlement History**

| 1. Where were you born? |  | | |
| --- | --- | --- | --- |
| 1. What year did you move to your current boma? |  | | |
| 1. Why did you move to your current boma? |  | | |
| 1. Where did you live prior to your current boma? |  | | |
| 1. Have you ever had to move due to a conservancy?   If YES, which conservancy?  What year?  Where did you move to?  Did you move to a relative/clan/friends boma?  If YES, who? *(specify relationship)* | YES | | NO |
|  |  |  |  |
|  |  |  |  |
|  |  |  |  |
|  | YES | NO | |
|  |  |  |  |

1. **Land ownership**
2. Are you on the Koyiaki Group Ranch register? YES/NO
3. Are you another Group Ranch register? YES/NO If YES, which one? ________________
4. Do you own the land that your boma is on? YES/NO ***If NO, go to question G***
5. If YES, how much land do you own? Size_____________ acres
6. How did you acquire the land?

|  | | Which year? |  | | | Title deed? | |
| --- | --- | --- | --- | --- | --- | --- | --- |
| Allocated at sub-division |  |  |  |  |  | YES | NO |
| Inherited* |  |  |  |  |  | Transfer? | |
| Bought |  |  | Cost? |  |  | YES | NO |
| Other (specify)_______________ |  |  |  | | | | |

**if inherited check and specify if land is now exclusively his/hers or if it is still with father, or if shared between brothers/wives – how many brothers/wives?*

1. How would you rate your land in terms of the following factors?

| Factor | Very poor | Poor | Ok | Good | Very good |
| --- | --- | --- | --- | --- | --- |
| Quality of grass |  |  |  |  |  |
| Quantity of grass |  |  |  |  |  |
| Access to water |  |  |  |  |  |
| Proximity to salt lick |  |  |  |  |  |
| Tourism potential |  |  |  |  |  |

1. If NO, are you?

| Staying because of the owners goodwill |  |
| --- | --- |
| Staying because owner is a relative |  |
| Renting the land |  |
| Settled when land was communal |  |
| Other (*specify*)___________________ |  |

1. Do you have land elsewhere? YES/NO ***If respondent has no land at all, go to question L***

IF YES,

|  | | | | | | Title deed? | |
| --- | --- | --- | --- | --- | --- | --- | --- |
| Location 1) | Size (acres) | How acquired? | | Which year? |  | YES | NO |
|  |  |  |  |  |  | Transfer? | |
|  |  | Allocated at subdivision |  |  |  | YES | NO |
|  |  | Inherited* |  |  |  |  | |
|  |  | Bought |  |  | Cost? |  | |
|  |  | Other (*specify*)_________ |  |  |  | | |

|  | | | | | | Title deed? | |
| --- | --- | --- | --- | --- | --- | --- | --- |
| Location 2) | Size (acres) | How acquired? | | Which year? |  | YES | NO |
|  |  |  |  |  |  | Transfer? | |
|  |  | Allocated at subdivision |  |  |  | YES | NO |
|  |  | Inherited* |  |  |  |  | |
|  |  | Bought |  |  | Cost? |  | |
|  |  | Other (*specify*)_________ |  |  |  | | |

|  | | | | | | Title deed? | |
| --- | --- | --- | --- | --- | --- | --- | --- |
| Location 3) | Size (acres) | How acquired? | | Which year? |  | YES | NO |
|  |  |  |  |  |  | Transfer? | |
|  |  | Allocated at subdivision |  |  |  | YES | NO |
|  |  | Inherited* |  |  |  |  | |
|  |  | Bought |  |  | Cost? |  | |
|  |  | Other (*specify*)_________ |  |  |  | | |

|  | | | | | | Title deed? | |
| --- | --- | --- | --- | --- | --- | --- | --- |
| Location 4) | Size (acres) | How acquired? | | Which year? |  | YES | NO |
|  |  |  |  |  |  | Transfer? | |
|  |  | Allocated at subdivision |  |  |  | YES | NO |
|  |  | Inherited* |  |  |  |  | |
|  |  | Bought |  |  | Cost? |  | |
|  |  | Other (*specify*)_________ |  |  |  | | |

**if inherited check and specify if land is now exclusively his/hers or if it is still with father, or if shared between brothers/wives – how many brothers/wives?*

1. How would you rate this land in terms of the following factors?

Location 1_________________

| Factor | Very poor | Poor | Ok | Good | Very good |
| --- | --- | --- | --- | --- | --- |
| Quality of grass |  |  |  |  |  |
| Quantity of grass |  |  |  |  |  |
| Access to water |  |  |  |  |  |
| Proximity to salt lick |  |  |  |  |  |
| Tourism potential |  |  |  |  |  |

Location 2________________

| Factor | Very poor | Poor | Ok | Good | Very good |
| --- | --- | --- | --- | --- | --- |
| Quality of grass |  |  |  |  |  |
| Quantity of grass |  |  |  |  |  |
| Access to water |  |  |  |  |  |
| Proximity to salt lick |  |  |  |  |  |
| Tourism potential |  |  |  |  |  |

Location 3________________

| Factor | Very poor | Poor | Ok | Good | Very good |
| --- | --- | --- | --- | --- | --- |
| Quality of grass |  |  |  |  |  |
| Quantity of grass |  |  |  |  |  |
| Access to water |  |  |  |  |  |
| Proximity to salt lick |  |  |  |  |  |
| Tourism potential |  |  |  |  |  |

Location 4_________________

| Factor | Very poor | Poor | Ok | Good | Very good |
| --- | --- | --- | --- | --- | --- |
| Quality of grass |  |  |  |  |  |
| Quantity of grass |  |  |  |  |  |
| Access to water |  |  |  |  |  |
| Proximity to salt lick |  |  |  |  |  |
| Tourism potential |  |  |  |  |  |

1. Is any piece of your land fenced? YES/NO

If YES,

| Location | Type of fence | What area is fenced? (*Approx acres)* | Reason for fencing |
| --- | --- | --- | --- |
|  |  |  |  |
|  |  |  |  |

1. Do you plan to fence any of your land in the next 1 year? YES/NO

If YES, location_______________ Area to be fenced____________Reason for fencing___________________

1. On any piece of land you live on, or own, has anyone moved there because of a conservancy? YES/NO

If YES, who? _____________________________________(*be specific on number of relatives/friends/clan etc)*

From which conservancy? ___________________________

To which place?_____________________________

What year? ______________________________________

1. Have you sold any land in the last 5 years? YES/NO

If YES, where? __________________When? _________________ Acres? __________Cost?____________

1. Do you plan to BUY any land in the next 1 year? YES/NO

If YES, where?______________________ When? _________________________ Acres?________________

1. Do you plan to SELL any land in the next 1 year? YES/NO

If YES, where?______________________ When? _________________________ Acres?________________

1. Does anyone else in your *olmarei* own land? YES/NO

Who?______________________________

Where?____________________________

Acres?_____________________________

How acquired? ______________________________________What year?____________________________.

1. **Household features/livestock ownership**
2. Do you or any members of your *olmarei* own any of the following?

*Indicate Y or N for each column*

| Phone |  |
| --- | --- |
| Radio |  |
| TV |  |
| Bicycle |  |
| Motorbike |  |
| Car |  |
| Tractor |  |
| Solar panel |  |

1. How many houses does the *olmarei* utilize and what is the roofing and wall material for each?

***Wall:*** *Mud=1; mabati=2; wire/stones/plaster=3; bricks=4; planks=5; other=6 (specify)*

***Roof:*** *Mud=1; mabati=2; grass=3; other=4 (specify)*

| **House no.** | **Wall** | **Roof** |
| --- | --- | --- |
|  |  |  |
|  |  |  |
|  |  |  |
|  |  |  |
|  |  |  |
|  |  |  |
|  |  |  |

1. How many livestock does the *olmarei* currently own?

*(Enter both total cattle number and then numbers for each category and cross-check)*

| **Animals** | | **Number** |
| --- | --- | --- |
| Cattle | Bulls |  |
|  | Steers |  |
|  | Lactating females |  |
|  | Non-lactating females |  |
|  | Heifer |  |
|  | Calves |  |
| Sheep | |  |
| Goats | |  |
| Donkeys | |  |

1. Type of fence for Cattle _____________ ***Code:*** *Maasai traditional=1; poles=2; mix traditional/poles=3;*

*mix traditional/wire=4; other=5 (specify)*

1. Type of fence for shoats _____________

***NOTE****: From here onwards remember to specify the time period when asking these questions and be precise about the months. I.e. For one year: from July 2009-June 2010, and for 6 months: from Jan-June 2010.*

1. **Livestock movements/grazing**
2. Where are your livestock currently?

| Location | No of Cattle | No of Shoats |
| --- | --- | --- |
| Home |  |  |
| Other *(specify where and with who?)..………………………...* |  |  |
|  |  |  |

1. Where did you graze your animals in the recent drought? Cattle 1) ________________2) ________________

Shoats1) _______________ 2) ________________

1. In the last one year have you herded your livestock together with other herds? YES/NO

If YES, whose herds? ___________________________

1. In the last one year, have you grazed your livestock inside the reserve? YES/NO

If YES, how often?

| Most days |  |
| --- | --- |
| 1-2 x per month |  |
| In dry times only |  |
| In wet season only |  |
| Other (specify)______________ |  |

Which months? __________________________________ Day / Night

1. Have you had any conflict with rangers of the reserve in the last one year? YES/NO

If YES, how many times?___________________

Have you paid a fine? YES/NO How many times in the last year? __________________

Total spent on fines by the *olmarei* in the last year?_____________KSH

*(taking into account if the cost is split between other herds)*

1. In the last one year, have you grazed your livestock inside any conservancy? YES/NO

If YES, which one/s?

| **How often?** | | OOC | | Motorogi | | Mara North | | Naboisho | |
| --- | --- | --- | --- | --- | --- | --- | --- | --- | --- |
| Most days | | |  | |  | |  | |  |
| 1-2 x per month | | |  | |  | |  | |  |
| In dry times only | | |  | |  | |  | |  |
| In wet season only | | |  | |  | |  | |  |
| Other_______________ | | |  | |  | |  | |  |
|  |  | | | | | | | | |
| Which months? | | |  | |  | |  | |  |
| Day or Night? | | | Day / Night | | Day / Night | | Day / Night | | Day / Night |

1. Have you had any conflict with rangers of any conservancy in the last one year? YES/NO

If YES, which conservancy/s? _________________________How many times?________________________

Have you paid a fine? YES/NO How many times in the last year?__________________

Total spent on fines by the *olmarei* in the last year?_____________KSH

*(taking into account if the cost is split between other herds)*

1. Did you graze your livestock inside any conservancy before it was set up? YES/NO

If YES, which conservancy/s? (*circle the correct choice/s*)

| OOC | Motorogi | Mara North | Naboisho |
| --- | --- | --- | --- |

1. Do you consider the conservancy/s to be a benefit for your livestock or a cost? BENEFIT / COST

Please explain your answer__________________________________________________________________

________________________________________________________________________________________

________________________________________________________________________________________

1. **Livestock In/Out:**
2. In the last **6 months** how many times have you sold or otherwise given out livestock? *(e.g. slaughter, gift, debt, exchange)*

| Type of animal sold/out? | How many sold/out? | Reason out:  *(1=sold; 2=gift; 3=debt; 4=exchange; 5=slaughter)* | If sold, why? | If sold, price? (KSH) *(indicate price for each animal)* |
| --- | --- | --- | --- | --- |
|  |  |  |  |  |
|  |  |  |  |  |
|  |  |  |  |  |
|  |  |  |  |  |
|  |  |  |  |  |
|  |  |  |  |  |
|  |  |  |  |  |
|  |  |  |  |  |
|  |  |  |  |  |
|  |  |  |  |  |
|  |  |  |  |  |

1. In the last **6 months** how many times have you bought or otherwise received livestock? *(e.g. gift, exchange; debt repaid?)*

| Type of animal bought/received? | How many bought/received? | Reason in:  *(1=bought; 2=gift; 3=debt repaid; 4=exchange)* | If bought, price? (KSH)  *(indicate price for each animal)* |
| --- | --- | --- | --- |
|  |  |  |  |
|  |  |  |  |
|  |  |  |  |
|  |  |  |  |
|  |  |  |  |
|  |  |  |  |
|  |  |  |  |
|  |  |  |  |
|  |  |  |  |

1. **Livestock losses**
2. Can you estimate how many livestock you have lost to drought and disease in the last one year?

| Period | Cattle | | | Sheep | | Goats | |
| --- | --- | --- | --- | --- | --- | --- | --- |
|  | Drought | Disease | Which disease? | Drought | Disease | Drought | Disease |
| *May-July 2010* |  |  |  |  |  |  |  |
| Jan-April 2010 |  |  |  |  |  |  |  |
| Sept-Dec 2009 |  |  |  |  |  |  |  |
| May-Aug 2009 |  |  |  |  |  |  |  |

1. How many livestock have you lost to predators in the last one year, and were you compensated for any?

|  | Cattle | Sheep | Goats | Compensation/  Ksh per animal |
| --- | --- | --- | --- | --- |
| Lion |  |  |  |  |
| Leopard |  |  |  |  |
| Hyena |  |  |  |  |
| Other (*specify*)__________ |  |  |  |  |

1. **Livestock products**
2. Have you sold any livestock products in the last one year?

|  | YES/NO | Which months did you sell? | How many sold per day/week/month? | Sold where? | Price per quantity sold? |
| --- | --- | --- | --- | --- | --- |
| Milk |  |  |  |  |  |
| Hides or Skins |  |  |  |  |  |
| Other (*specify*)___________ |  |  |  |  |  |

Bottle = ___________ ML

1. Have you had to buy milk for your *olmarei* in the last one year? YES/NO

If YES, which months?_________________________________________

How much did you buy (per day)? _______________________________Litres/ML/KG

How much did you spend per quantity bought? ________________________________KSH

1. **Conservancy involvement**
2. Are you or a member of your *olmarei* currently a member of a conservancy? YES/NO

***If NO, go to question U***

If, YES:

| B | Which *olmarei* member? | 1) | | 2) | | 3) | |
| --- | --- | --- | --- | --- | --- | --- | --- |
| C | Which conservancy/s | 1) | | 2) | | 3) | |
| D | What year did you/they join? |  | |  | |  | |
| E | How much land do you/they have in the conservancy?*(acres)* |  | |  | |  | |
| F | Are you/they on the committee or management staff?  If YES, what position? | YES | NO | YES | NO | YES | NO |
|  |  |  |  |  |  |  |  |
| G | How much did you/they receive from the conservancy per month in the last year? | Payment amount (KSH) | | Payment amount (KSH) | | Payment amount (KSH) | |
|  | July 2010 |  | |  | |  | |
|  | June 2010 |  | |  | |  | |
|  | May 2010 |  | |  | |  | |
|  | April 2010 |  | |  | |  | |
|  | March 2010 |  | |  | |  | |
|  | Feb 2009 |  | |  | |  | |
|  | Jan 2009 |  | |  | |  | |
|  | Dec 2009 |  | |  | |  | |
|  | Nov 2009 |  | |  | |  | |
|  | Oct 2009 |  | |  | |  | |
|  | Sept 2009 |  | |  | |  | |
|  | Aug 2009 |  | |  | |  | |

1. What is the main reason you joined the conservancy/s?

| For income |  |
| --- | --- |
| For wildlife conservation |  |
| Pressure from the community |  |
| For dry season grazing |  |
| Other (*Specify*) |  |

1. Do you split your payment with anyone as payment for residence on someone else’s land? YES/NO

If YES, who? _______________________________How is it split? __________________________________

1. Do you consider the current payment amount to be sufficient? YES/NO

If NO, how much would you like it to be for a full 150 acre parcel? KSH____________

1. Are you happy with the current system of payment to the bank? YES/NO

If NO, what mode of payment would you prefer? _________________

*(****Code****: 1=cash, 2=cheque, 3=MPESA, 4=non-cash (e.g. food), 5=other)*

1. Into whose bank account is the money deposited?

| Household head account |  |
| --- | --- |
| A joint account with wife/s |  |
| A friend’s account |  |
| A relative’s account. Who? ____________ |  |
| Other (*specify*)_______________________ |  |

1. How often do you travel to the bank to withdraw your conservancy payment?

| Twice a month |  |
| --- | --- |
| Once a month |  |
| Every 2 months |  |
| Every 3-6 months |  |
| Other (*specify*)___________________ |  |

1. In the last 3 months what have you spent your lease payment on? Please rank 1, 2 and 3.

| **Expense** | **Rank** |
| --- | --- |
| Education |  |
| Basic needs (e.g. food, clothes) |  |
| Livestock purchases |  |
| Veterinary costs |  |
| Grazing purchases |  |
| Health expenses |  |
| Grazing fines |  |
| Hired herder |  |
| Other(*specify*)____________ |  |

1. Apart from the payment do you receive any other benefits from the conservancy/s? YES/NO

| 1. |
| --- |
| 2. |
| 3 |

1. Have you experienced any costs or problems due to the conservancy/s? YES/NO

| 1. |
| --- |
| 2. |
| 3 |

1. How often do you attend meetings about the conservancy/s?

| Weekly |  |
| --- | --- |
| Monthly |  |
| Quarterly |  |
| Yearly |  |
| Other (*specify*)______________ |  |

1. Do you feel you had adequate information about the conservancy before joining? YES/NO

If NO, what would you have liked to know before joining the conservancy?

| 1. |
| --- |
| 2. |
| 3 |

1. Are you happy with the current duration of the lease contract? YES/NO

If NO, how long would you like it to be? _____________ Years

1. Have you signed the 15 year contract? YES / NO / UNSURE –depends on…………….…………

***For NON-members***

1. Do you have land within a conservancy area but have not joined the conservancy? YES/NO

If YES, why have you not joined? _______________________________________________________________

__________________________________________________________________________________________

1. Were you involved in any conservancy or association before? YES/NO

| Which conservancy/association? | For what time period? | Why did you stop? |
| --- | --- | --- |
|  |  |  |
|  |  |  |

1. Do you receive conservancy payment as rent from someone who is a member of a conservancy and is living on your land? YES/NO

If YES, who? _________________________________

From which conservancy?_________________________

Since when?_________________________________

How is the payment split? _______________________

1. Do you experience any benefits due to a conservancy? YES/NO

| 1. |
| --- |
| 2. |
| 3 |

1. Do you experience any costs or problems due to a conservancy? YES/NO

| 1. |
| --- |
| 2. |
| 3 |

***For BOTH members and non-members***

1. What do you think is good about the conservancy?

| 1. |
| --- |
| 2. |
| 3 |
| 4. |

1. What do you think is bad about the conservancy?

| 1. |
| --- |
| 2. |
| 3 |
| 4. |

AB. Do you think conservancy members did better than non-members in the recent drought, or no difference?

| Members did better |  | Reason |
| --- | --- | --- |
| Non-members did better |  |
| No difference |  |

1. **Cultivation**
2. In the last one year, have you or a member of your *olmarei* been involved in cultivation, here or elsewhere? YES/NO

***If NO, go to question H***

1. If YES, which *olmarei* member/s? _______________________
2. In what way?

| Cultivating own plot |  |
| --- | --- |
| Cultivating on community land |  |
| Renting a plot from someone |  |
| Renting a plot to someone |  |
| Other (*specify*)_____________________ |  |

1. How much did the *olmarei* cultivate, harvest, consume, and was any sold?

| Location | Crop | No. of acres | Water type-rain fed or irrigated | Amount Harvested? Kg/bags | Amount consumed? Kg/bags | Amount sold? Kg/bags and selling price | Sold where? |
| --- | --- | --- | --- | --- | --- | --- | --- |
|  |  |  |  |  |  |  |  |
|  |  |  |  |  |  |  |  |
|  |  |  |  |  |  |  |  |
|  |  |  |  |  |  |  |  |

1. Of the crops consumed, for how long did this feed the o*lmarei*?_______________________________
2. Are there crops now in the ground which have not yet been harvested? YES/NO

| Location | Crop | No. of acres | Water type – rain fed/ irrigated |
| --- | --- | --- | --- |
|  |  |  |  |
|  |  |  |  |
|  |  |  |  |
|  |  |  |  |

1. What year did the o*lmarei* begin cultivation? __________________
2. If NO:

| 1. Why does the *olmarei* not cultivate? |  | | |
| --- | --- | --- | --- |
| 1. Has the *olmarei* cultivated in previous years?   Where did you cultivate?  When did you cultivate?  Why did you stop? | YES | | NO |
|  | | |
|  | | |
|  | | |
| 1. Do you have plans to cultivate in the future?   Where?  When?  What crops? | YES | NO | |
|  | | |
|  | | |
|  | | |

1. **Off-Farm Activities**

Can you give us information about other sources of income of any member of the *olmarei* in the last one year (including those that may have stopped) and describe what they are/were doing:

| Profession/activity*  *(specify below)* | No. of persons | Relation-ship to *olmarei* head** | Frequency of contributions to the *olmarei:*  *(1= occasional; 2=weekly; 3=monthly; 4=daily)* | Average amount coming into the household per contribution? | When did the activity begin? | If the activity has now stopped, when did it stop? | *Why* did it stop? | Notes |
| --- | --- | --- | --- | --- | --- | --- | --- | --- |
| Tourism/conservation employee  *(what? where?)* |  |  |  |  |  |  |  |  |
| Teacher |  |  |  |  |  |  |  |  |
| Campsite fees  *(where*?) |  |  |  |  |  |  |  |  |
| Craft sales  *(where?)* |  |  |  |  |  |  |  |  |
| Remittances *(i.e. money coming to olmarei from person elsewhere)* |  |  |  |  |  |  |  |  |
| Livestock trader:  (N*o. and type of animals per week on average)*: |  |  |  |  |  |  |  |  |
| Business person or trader: (*Specify e.g. what shop or trade?)* |  |  |  |  |  |  |  |  |
| Transport business (*specify):* |  |  |  |  |  |  |  |  |
| Other activity (specify): |  |  |  |  |  |  |  |  |
| Other activity *(specify)*: |  |  |  |  |  |  |  |  |

** Probe the respondent about different activities e.g. local brewing, ranger, casual labour, sale of sand/stones, research work, Maasai singing/dancing, others.....*

** *1=household head; 2=spouse; 3=son; 4=daughter; 5=mother; 6=other (specify)*

# Household Expenditure

Can you estimate your *olmarei* expenses for the last 1 month on the following items?

| **Expense** | **KSH** |
| --- | --- |
| School fees (per term) |  |
| Livestock purchases |  |
| Veterinary costs |  |
| Water purchases |  |
| Grazing purchases |  |
| Crop farming expenses |  |
| Health expenses |  |
| Basic needs (food, clothes) |  |
| Transport costs |  |
| Hired herder |  |
| Grazing fines |  |
| Other (*specify*)________________ |  |

1. **Livelihood importance**

What do you consider to be the most important livelihood activity for your overall *olmarei* welfare?

Please provide 1^st^, 2^nd^ and 3^rd^ rankings of the following livelihood activities.

| **Activity** | **Rank** |
| --- | --- |
| Livestock keeping |  |
| Cultivation |  |
| Conservancy member |  |
| Business |  |
| Wage/job |  |
| Campsite fee |  |
| Craft sales |  |
| Livestock trader |  |
| Remittances |  |
| Other (*specify*)________________ |  |
