# Supplementary material for: Trade-offs for climate-resilient pastoral livelihoods in wildlife conservancies in the Mara ecosystem, Kenya
Source: Pastoralism. 2017 May 23;7(1):10. doi: 10.1186/s13570-017-0085-1 (PMC6991982; doi:10.1186/s13570-017-0085-1)
Supplement: Supplementary file 2 — Table showing the dates and seasons of the 60 aerial surveys conducted in the Mara ecosystem between 1977 and 2014 by the Directorate of Resource Surveys and Remote Sensing (DRSRS). (DOCX 15 kb) [file 13570_2017_85_MOESM2_ESM.docx]

Table S1. Showing the dates and seasons of the 60 aerial surveys conducted in the Mara Ecosystem between 1977 and 2014 by the Directorate of Resource Surveys and Remote Sensing (DRSRS)

| **Ecosystem** | **Census ID** | **Date of survey** | **StartDate** | **EndDate** | **Season** |
| --- | --- | --- | --- | --- | --- |
| Masai Mara Ecosystem | 7701 | 29/01/77 | 27/01/77 | 29/01/77 | Wet |
| Masai Mara Ecosystem | 7706 | 30/11/77 | 25/11/77 | 30/11/77 | Wet |
| Masai Mara Ecosystem | 7801 | 08/04/78 | 15/03/78 | 08/04/78 | Wet |
| Masai Mara Ecosystem | 7806 | 11/12/78 | 11/12/78 | 11/12/78 | Wet |
| Masai Mara Ecosystem | 7901 | 26/01/79 | 23/01/79 | 26/01/79 | Wet |
| Masai Mara Ecosystem | 7904 | 28/02/79 | 26/02/79 | 28/02/79 | Wet |
| Masai Mara Ecosystem | 7906 | 30/03/79 | 28/03/79 | 30/03/79 | Wet |
| Masai Mara Ecosystem | 7908 | 28/04/79 | 26/04/79 | 28/04/79 | Wet |
| Masai Mara Ecosystem | 7909 | 07/06/79 | 06/06/79 | 07/06/79 | Wet |
| Masai Mara Ecosystem | 7910 | 05/07/79 | 02/07/79 | 05/07/79 | Dry |
| Masai Mara Ecosystem | 7912 | 03/08/79 | 30/07/79 | 03/08/79 | Dry |
| Masai Mara Ecosystem | 7913 | 28/08/79 | 26/08/79 | 28/08/79 | Dry |
| Masai Mara Ecosystem | 7917 | 09/10/79 | 05/10/79 | 09/10/79 | Dry |
| Masai Mara Ecosystem | 7919 | 02/11/79 | 30/10/79 | 02/11/79 | Dry |
| Masai Mara Ecosystem | 7920 | 22/11/79 | 19/11/79 | 22/11/79 | Dry |
| Masai Mara Ecosystem | 8015 | 31/10/80 | 27/10/80 | 31/10/80 | Dry |
| Masai Mara Ecosystem | 8101 | 16/01/81 | 15/01/81 | 16/01/81 | Wet |
| Masai Mara Ecosystem | 8114 | 29/10/81 | 27/10/81 | 29/10/81 | Dry |
| Masai Mara Ecosystem | 8116 | 28/11/81 | 25/11/81 | 28/11/81 | Dry |
| Masai Mara Ecosystem | 8110 | 29/08/81 | 27/08/81 | 29/08/81 | Dry |
| Masai Mara Ecosystem | 8201 | 22/02/82 | 21/02/82 | 22/02/82 | Wet |
| Masai Mara Ecosystem | 8206 | 25/05/82 | 24/05/82 | 25/05/82 | Wet |
| Masai Mara Ecosystem | 8207 | 01/09/82 | 30/08/82 | 01/09/82 | Dry |
| Masai Mara Ecosystem | 8209 | 09/12/82 | 06/12/82 | 09/12/82 | Wet |
| Masai Mara Ecosystem | 8301 | 14/02/83 | 03/02/83 | 14/02/83 | Wet |
| Masai Mara Ecosystem | 8307 | 01/08/83 | 29/07/83 | 01/08/83 | Dry |
| Masai Mara Ecosystem | 8311 | 03/10/83 | 30/09/83 | 03/10/83 | Dry |
| Masai Mara Ecosystem | 8315 | 04/12/83 | 01/12/83 | 04/12/83 | Wet |
| Masai Mara Ecosystem | 8401 | 23/01/84 | 21/01/84 | 23/01/84 | Wet |
| Masai Mara Ecosystem | 8408 | 16/08/84 | 15/08/84 | 16/08/84 | Dry |
| Masai Mara Ecosystem | 8505 | 26/04/85 | 24/04/85 | 26/04/85 | Wet |
| Masai Mara Ecosystem | 8512 | 01/12/85 | 28/11/85 | 01/12/85 | Wet |
| Masai Mara Ecosystem | 8607 | 08/05/86 | 08/05/86 | 08/05/86 | Wet |
| Masai Mara Ecosystem | 8608 | 15/08/86 | 10/08/86 | 15/08/86 | Dry |
| Masai Mara Ecosystem | 8615 | 12/11/86 | 08/11/86 | 12/11/86 | Wet |
| Masai Mara Ecosystem | 8706 | 28/04/87 | 24/04/87 | 28/04/87 | Wet |
| Masai Mara Ecosystem | 8902 | 15/05/89 | 12/05/89 | 15/05/89 | Wet |
| Masai Mara Ecosystem | 9003 | 16/08/90 | 13/08/90 | 16/08/90 | Dry |
| Masai Mara Ecosystem | 9102 | 21/04/91 | 20/04/91 | 21/04/91 | Wet |
| Masai Mara Ecosystem | 9103 | 20/08/91 | 12/08/91 | 20/08/91 | Dry |
| Masai Mara Ecosystem | 9202 | 23/03/92 | 14/03/92 | 23/03/92 | Wet |
| Masai Mara Ecosystem | 9204 | 24/08/92 | 19/08/92 | 24/08/92 | Dry |
| Masai Mara Ecosystem | 9306 | 12/11/93 | 03/11/93 | 12/11/93 | Dry |
| Masai Mara Ecosystem | 9402 | 25/05/94 | 15/05/94 | 25/05/94 | Wet |
| Masai Mara Ecosystem | 9604 | 08/08/96 | 27/07/96 | 08/08/96 | Dry |
| Masai Mara Ecosystem | 9703 | 31/05/97 | 28/05/97 | 31/05/97 | Wet |
| Masai Mara Ecosystem | 9706 | 26/08/97 | 19/08/97 | 26/08/97 | Dry |
| Masai Mara Ecosystem | 0004 | 06/11/00 | 21/10/00 | 06/11/00 | Dry |
| Masai Mara Ecosystem | 0204 | 07/12/02 | 01/12/02 | 07/12/02 | Wet |
| Masai Mara Ecosystem | 0403 | 30/05/04 | 18/05/04 | 30/05/04 | Wet |
| Masai Mara Ecosystem | 0502 | 13/05/05 | 01/05/05 | 13/05/05 | Wet |
| Masai Mara Ecosystem | 0503 | 13/09/05 | 02/09/05 | 13/09/05 | Dry |
| Masai Mara Ecosystem | 0702 | 23/05/07 | 17/05/07 | 23/05/07 | Wet |
| Masai Mara Ecosystem | 0803 | 02/11/08 | 23/10/08 | 02/11/08 | Dry |
| Masai Mara Ecosystem | 0903 | 26/10/09 | 17/10/09 | 26/10/09 | Dry |
| Masai Mara Ecosystem | 1004 | 25/10/10 | 10/10/10 | 25/10/10 | Dry |
| Masai Mara Ecosystem | 1105 | 13/10/11 | 01/10/11 | 13/10/11 | Dry |
| Masai Mara Ecosystem | 1205 | 01/11/12 | 27/10/12 | 01/11/12 | Dry |
| Masai Mara Ecosystem | 1303 | 30/11/13 | 26/11/13 | 30/11/13 | Dry |
| Masai Mara Ecosystem | 1403 | 15/09/14 | 01/09/14 | 15/09/14 | Dry |
